# Functional microRNA targetome undergoes degeneration-induced shift in the retina

**DOI:** 10.1186/s13024-021-00478-9

**Published:** 2021-08-31

**Authors:** Joshua A. Chu-Tan, Adrian V. Cioanca, Zhi-Ping Feng, Yvette Wooff, Ulrike Schumann, Riemke Aggio-Bruce, Hardip Patel, Matt Rutar, Katherine Hannan, Konstantin Panov, Jan Provis, Riccardo Natoli

**Affiliations:** 1 Eccles Institute of Neuroscience, The John Curtin School of Medical Research, College of Health and Medicine, The Australian National University, Acton, Canberra, ACT 2601 Australia; 2 The Australian National University Medical School, College of Health and Medicine, Canberra, ACT 2601 Australia; 3 The ANU Bioinformatics Consultancy, The John Curtin School of Medical Research, College of Health and Medicine, The Australian National University, Acton, Canberra, ACT 2601 Australia; 4 School of Biomedical Sciences, The University of Melbourne, Parkville, Victoria 3010 Australia; 5 Faculty of Science and Technology, University of Canberra, Bruce, ACT 2617 Australia; 6 ACRF Department of Cancer Biology and Therapeutics, The John Curtin School of Medical Research, College of Health and Medicine, The Australian National University, Acton, Canberra, ACT 2601 Australia; 7 School of Biological Sciences Queen’s University Belfast, Belfast, BT9 5DL Northern Ireland

**Keywords:** Retina, microRNA, mRNA, Retinal degeneration, Argonaute, HITS-CLIP, Transcriptome, Inflammation

## Abstract

**Background:**

MicroRNA (miRNA) play a significant role in the pathogenesis of complex neurodegenerative diseases including age-related macular degeneration (AMD), acting as post-transcriptional gene suppressors through their association with argonaute 2 (AGO2) - a key member of the RNA Induced Silencing Complex (RISC). Identifying the retinal miRNA/mRNA interactions in health and disease will provide important insight into the key pathways miRNA regulate in disease pathogenesis and may lead to potential therapeutic targets to mediate retinal degeneration.

**Methods:**

To identify the active miRnome targetome interactions in the healthy and degenerating retina, AGO2 HITS-CLIP was performed using a rodent model of photoreceptor degeneration. Analysis of publicly available single-cell RNA sequencing (scRNAseq) data was performed to identify the cellular location of AGO2 and key members of the microRNA targetome in the retina. AGO2 findings were verified by in situ hybridization (RNA) and immunohistochemistry (protein).

**Results:**

Analysis revealed a similar miRnome between healthy and damaged retinas, however, a shift in the active targetome was observed with an enrichment of miRNA involvement in inflammatory pathways. This shift was further demonstrated by a change in the seed binding regions of miR-124-3p, the most abundant retinal AGO2-bound miRNA, and has known roles in regulating retinal inflammation. Additionally, photoreceptor cluster miR-183/96/182 were all among the most highly abundant miRNA bound to AGO2. Following damage, AGO2 expression was localized to the inner retinal layers and more in the OLM than in healthy retinas, indicating a locational miRNA response to retinal damage.

**Conclusions:**

This study provides important insight into the alteration of miRNA regulatory activity that occurs as a response to retinal degeneration and explores the miRNA-mRNA targetome as a consequence of retinal degenerations. Further characterisation of these miRNA/mRNA interactions in the context of the degenerating retina may provide an important insight into the active role these miRNA may play in diseases such as AMD.

**Supplementary Information:**

The online version contains supplementary material available at 10.1186/s13024-021-00478-9.

## Background

MicroRNA (miRNA) are a class of small endogenous non-coding RNAs that act as post-transcriptional gene repressors, and have been implicated in the control of cellular and tissue homeostasis, development and biological pathway regulation [1]. miRNAs are incorporated into argonaute (AGO) proteins which then assemble to be part of the RNA-induced silencing complex (RISC) [2–7]. Once incorporated into RISC, miRNAs guide binding of AGO to the 3′ untranslated (3′ UTR) of the mRNA target(s) through recognition of the miRNA seed region (typically 6–8 nucleotides long, commonly referred to as 6-mer, 7-mer and 8-mer seed region) [8]. This process results in post-transcriptional gene silencing through translational repression or mRNA degradation [9, 10].

A single miRNA has the ability to target hundreds of mRNAs, often all working in similar biological pathways, thus allowing for the mapping and identification of specific “miRNA networks” [11]. Due to their selective targeting ability, miRNAs have emerged as key orchestrators of the mammalian transcriptome. As such, their dysregulation has been implicated in the pathogenesis of multiple inflammatory diseases, cancers, neurological disorders as well as retinal degenerative diseases including age-related macular degeneration (AMD) [12–16], a disorder that specifically affects the central vision due to progressive damage to the light-sensing photoreceptor cells in the macular region. Retinal degenerations such as AMD have complex and multi-faceted etiology, with causal links to the pathogenesis of how retinal degeneration develops in AMD remaining elusive [17–22].

Multiple miRNAs have been postulated to play a role in both retinal homeostasis and pathogenesis including in AMD [14, 15, 23–30]. An important retinal miRNA subset is the “photoreceptor cluster,” miR-182/96/183, which together have been established as key in the development of the light-sensing photoreceptors of the retina. Another example is miR-124, which has been heavily studied in the central nervous system (CNS) and is the most highly abundant miRNA in the brain [31–34]. In the retina, miR-124 is expressed in the photoreceptor neurons and has been documented to play an anti-inflammatory role in neuronal tissue with reduced miR-124 in the retinal microglia cells leading to an increase in the activation of these cells [14]. However, a comprehensive list of their targets has, currently, only been largely predicted and elucidation of their functional targets would provide crucial insight into their specific roles in the retina during health and disease.

In order to further advance our understanding of the molecular roles that miRNAs play in the normal and degenerating retina, we performed high-throughput sequencing following cross-linking immunoprecipitation (HITS-CLIP) [35] in the retina. This enables high confidence validation of miRNA targets, which has historically been problematic when based on in silico predictions [36–40]. AGO2, as a core component of the RISC complex, has been implicated in the biogenesis and maturation of miRNAs, in addition to directly binding mature miRNAs for mRNA target repression [41]. Importantly, AGO2 CLIP enables the identification of only RISC-complexed, biologically functional miRNAs and their resultant targetome [36–40]. This is afforded from crosslinking RNA binding proteins (AGO2) with miRNAs and mRNAs, immunoprecipitating AGO2 proteins and subsequently sequencing the associated RNA.

In the study presented here, we revealed that the majority of the retinal AGO2-bound miRnome was comprised of only a small miRNA subset, with miR-124-3p being highly represented. While the retinal AGO2-bound miRnome did not differ in expression between healthy and damaged retinas, we identified an altered AGO2-bound mRNA profile, representing a shift in the targetome following retinal damage. This was associated with a dynamic change in seed region binding of miR-124-3p. Finally, we showed that upon retinal damage AGO2 accumulated in the inner retinal layers and colocalized more strongly with the Müller glia, which suggests a major shift in miRNA activity in these cells. Together these results provide mechanistic insight into the effects of degeneration on the miRNA-mRNA regulatory networks in the retina.

## Methods

### Animal experiments

All animal experiments were conducted in accordance with the ARVO Statement for Use of Animals in Ophthalmic and Vision Research and with approval from the ANU Animal Experimentation Ethics Committee (Ethics ID: A2014/56). C57BL/6 J mice aged between P60–80 and devoid of the RPE^65450Met^ and Crb1^rd8^ mutations were used for experiments. Mice were born and reared in individually vented cages in cyclic dim light conditions (12 h at 5 lx, 12 h at 0 lx). Müller glia reporter animals were derived by crossing the PDGFRa-Cre transgenic mice (strain ID: #013148; The Jackson Laboratory) with a Gt (ROSA)26Sortm14 floxed RFP reporter strain (ID: 007914, The Jackson Laboratory) maintained on the C57BL/6 J background and screened for the RPE^65450Met^ and Crb1^rd8^ mutations.

### Photo-oxidative damage

Photo-oxidative damage (PD) was used to mimic the pathogenesis of atrophic AMD in mice as described previously [42]. Animals were placed into perspex boxes coated with a reflective interior surface and exposed to 100 K lux white light from light-emitting diodes (LED). Exposure was continuous for a period of 5 days with food and water ad libitum. Animals were administered pupil dilator (Minims® atropine sulphate 1% w/v; Bausch and Lomb) to both eyes twice a day during the course of the damage paradigm. Dim-reared (dim) animals maintained in normal cyclic light rearing conditions were used as control.

### Tissue collection and preparation

Both dim and PD animals were culled by CO_2_ exposure. For retinal extraction, eyes were dissected using an ophthalmic knife, cut across the anterior surface. The lens was removed, the retina extracted using curved forceps and immediately immersed in RNAlater (Thermo Fisher Scientific, Waltham, MA) for overnight storage at − 4 °C then transferred to − 80 °C until needed. Six retinas from three animals were pooled for a single biological replicate, *n* = 4 biological replicate samples for both control and damaged retina were used for experimentation.

### Total RNA isolation from retinas

Total RNA was purified by acid phenol/chloroform lysis followed by the enrichment and purification of the aqueous phase for long and short RNA using the *mir*Vana™ Total RNA Isolation Kit (Thermo Fisher Scientific) according to manufacturer’s instructions. This yielded two samples: 1) small RNAs isolated from AGO2:miRNA and AGO2:miRNA:mRNA complexes; 2) mRNA isolated from AGO2:miRNA:mRNA complexes. RNA concentration was determined using a Qubit 4 Fluorometer (Thermo Fisher Scientific) and RNA quality was measured on a 2100 Bioanalyser using an RNA 6000 Nano Assay (Agilent Technologies, Santa Clara, CA). Only samples with an RNA integrity number (RIN) greater than 9.0 were carried forward to library preparation and high-throughput sequencing.

### AGO2 HITS-CLIP

Retinas were diced to a fine suspension in ice-cold PBS and then UV irradiated three times at 400 mJ/cm^2^ (Stratalinker) whilst swirling suspension between each irradiation to keep cold and ensure maximal surface exposure. Cross-linked tissue was either used directly or flash-frozen in liquid N_2_ and stored at − 80 °C. AGO2 HITS-CLIP was performed using a protocol adapted from Moore et al., 2014 [43]. All buffers were made fresh based on this aforementioned protocol [43]. Antibody-loaded beads were prepared as follows. Protein A Dynabeads™ (Thermo Fisher Scientific) were washed three times in 1 ml Bead Wash Buffer (BWB). The beads were suspended in BWB with 50 μg of rabbit anti-mouse IgG bridging antibody (Jackson ImmunoResearch, West Grove, PA) and rotated end over end at room temperature for 30 min, then washed again with BWB. IgG-loaded beads were then suspended in BWB, incubated with 4 μl Anti-pan Ago antibody (clone 2A8; Millipore, Burlington, MA), incubated at RT and washed as described above. Antibody-loaded beads were washed three times with 1xPXL buffer ensuring that beads are fully resuspended with each wash.

CLIP was performed as follows. Cross-linked retinal tissue was suspended in 1xPXL and incubated on ice for 10 min before lysis by gentle mechanical disruption using sterile pestles. 30 μl of RQ1 DNase (Promega, Madison, WI) was added and lysate incubated at 37 °C for 5 min whilst rotating at 1000 RPM. Lysates were incubated with 10 μl of 1x RNaseA (Affymetrix, Santa Clara, CA) per 1 ml of lysate and incubated at 37 °C for 5 min. Subsequently, lysates were kept ice-cold to minimize further RNase digestion. Lysates were centrifuged at 16,000x g for 40 min at 4 °C. The supernatant was collected and incubated with the antibody-loaded beads for 2 h at 4 °C whilst rotating end over end. The supernatant was removed and beads washed in a series of stringent wash steps: three times in 1xPXL; twice in high-salt buffer; twice in high-stringency buffer; 2 times in low-salt buffer; and twice in 1xPNK buffer.

To demonstrate the presence of RNA in AGO2 CLIP complexes, 3′ end-labeling followed by gel electrophoresis (SDS-PAGE) was performed. Residual PNK buffer was removed and beads resuspended in desphosphorylation master mix by gentle vortexing. The beads were incubated at 37 °C for 20 min, whilst shaking at 1000 RPM for 15 s every 2 min. Beads were washed once with 1xPNK (polynucleotide kinase) buffer, once with 1xPNK supplemented with EGTA and then twice with 1xPNK buffer. 3′ linker ligation master mix was added to the beads and incubated. Beads were washed with high-salt buffer, twice with 1xPNK buffer and then three times with 1xPNK/EGTA buffer. Residual buffer was removed, beads resuspended in 1xLDS sample-loading buffer (Thermo Fisher Scientific) and incubated at 70 °C for 15 min, whilst shaking at 1000 RPM. Beads were collected by centrifugation and the supernatant loaded onto a Novex NuPAGE™ Bis-Tris 8% gel (Invitrogen, Carlsbad, CA). Proteins were separated at 175 V for 3–4 h at 4 °C in 1x NuPAGE running buffer (Invitrogen) and then transferred to nitrocellulose membrane (BioRad) for 1 h at 90 V in 1 x NuPAGE transfer buffer (Invitrogen) containing 10% methanol. The membrane was rinsed in PBS, placed in a phosphorimage cassette and exposed to film (GE Healthcare, Chicago, IL) for 24 h. The film was imaged using a Phosphorimager (Typhoon FLA 9500, GE Healthcare). This confirmed the specific immunoprecipitation of AGO2-bound retinal miRNAs and mRNAs, visible as two distinct populations: 1) binary AGO2:miRNA complexes (110 kDa); 2) ternary AGO2:miRNA:mRNA complexes (“smear” at 130 kDa) (Chi et al., 2009, Moore et al., 2014) (Fig. 2B).

To maximise RNA yield, gel extraction of AGO2:RNA complexes was omitted. For RNA recovery following CLIP, bead-bound AGO2 was digested with 4 mg/ml of proteinase K (Roche Diagnostics). Contamination was controlled for with size enrichment of miRNA and mRNA populations within the AGO2-bound cleaved range [43]. Total RNA was purified with small RNA and mRNA fractions separated as aforementioned.

We exposed C57BL/6 J WT mice to 5 days photo-oxidative damage and isolated retinas were run through the AGO2 HITS-CLIP protocol (Fig. 2A). Retinal cell suspensions were UV cross-linked, subjected to limited RNase digestions, before AGO2 protein complexes were immunoprecipitation using the Anti-pan Ago antibody (clone 2A8; Millipore, Burlington, MA). This is an AGO2 specific antibody as used in the original AGO2 HITS-CLIP publication [43]. Captured complexes were washed and RNA released by proteinase K digestion.

### High-throughput sequencing (HTS) and bioinformatics

All library preparation and HTS was performed by the Biomolecular Research Facility (JCSMR, ANU). Sequencing libraries were prepared using either the CATS Small RNA-seq Kit (for miRNA samples) or the CATS RNA-seq Kit (for mRNA samples) according to manufacturer’s instructions (Diagenode, Denville, NJ). Libraries were sequenced on an Illumina HiSeq 2500, acquiring single-end reads of 51 bp for the global miRNA and HITS-CLIP experiments or 76 bp for the global mRNA experiment. Raw reads were processed with *cutadapt* to trim adaptors, artefact bases and barcodes. FASTQC sequence quality analysis were performed before and after cleaning. The average clean read length was 20 nt and 45 nt for miRNA and mRNA libraries, respectively.

Global high-throughput sequencing (HTS) of retinal miRNAs and mRNAs was performed to analyze overall changes in the retina between dim and 5 days PD mouse retinas. 47–71 million cleaned reads were obtained, reads aligned to the mm10 mouse genome and annotated according to RefSeq (Table S1). 3–9 million and reads were obtained and aligned to the mature mouse miRNA sequences downloaded from miRbase (Table S2).

Clean mRNA reads were aligned to the mm10 genome sequence with Burrows-Wheeler Aligner, *bwa-mem* [44] and *samtools* [45] was used for sorting and statistical analysis. Mapping statistics are shown in Table S1. Mapped reads were summarized using FeatureCounts [46] and annotated based on the genomic coordinates provided by Refseq. Trimmed Mean of M-values (TMM) [47–49] was used for normalization. Differential expression was analysed using the *voom-limma* package [47] with a linear model fit and Bayes’ adjustment of *p*-values to account for type I errors and multiple comparisons. Significance was determined as adjusted *P* < 0.05.

Mouse mature miRNA sequences were downloaded from miRbase [44, 50] and clean reads were separately aligned first to mouse mature miRNA and pri-miRNA sequences then to mm10 mouse reference genome using *bwa-aln* [44]. *awk*, *samtools* [45] and FeatureCounts [46] were used for count sorting and summary. Mapping statistics are shown in Table S1. Alignments were summarized using a Unix shell script (*awk* and *grep*) for reads with mapping quality above 20. The miRNAs were ranked based on their mean expression level. TMM method was [47–49] used for normalization and the voom-limma package with sample quality weights was used for detecting differentially expressed genes. For a robust differential expression analysis, only miRNAs with CPM > 10 in at least four out of the eight samples were used. Hierarchical clustering was performed using the *heatmap.2* function on R against the top 80 differentially expressed miRNA in the global database.

### Enrichment analysis

For peak calling, the mm10 genome alignment files were converted to the bed format and peaks called using Homer (http://homer.ucsd.edu/homer/), for PD and dim groups separately. Peak findings were analyzed using Factor styles which aims to identify the precise location of nucleic acid to protein contact. We used a fixed width peak size of 75 bp which was automatically estimated from the Tag Autocorrelation. The peaks were annotated using Homer (annotatePeaks.pl), extracted and separated into different regions (exon, intron, transcription termination site (TTS), ncRNA, miRNA, 5′ UTR, 3′ UTR). 3′ UTR peaks were searched for enriched sequence motifs using Homer (findMotifs.pl). The significantly expressed miRNAs and their targets were matched based on the motifs, their expression levels, and cross-referenced with our results from global miRNA and mRNA experiments.

For pathway enrichment, mRNA targets of the top 20 miRNA were identified by TargetScan [8] and miRNet [51]. These targets were batch-filtered and cross-referenced with the AGO HITS-CLIP mRNA data to yield functional mRNA targets. Enriched biological gene ontology processes were determined using Gorilla [52]. *P*-values were adjusted for multiple comparisons using the Benjamini-Hochberg method with terms with > 3 genes and *P* < 0.05 were deemed significantly. REVIGO was used for visualization of term clustering. Enrichment analysis was conducted for both up-regulated and down-regulated mRNA datasets. For in-depth pathway analysis, GSEA was performed with *camera* [53] against the gene sets listed in the Hallmark and C2 pathways from the Molecular Database (MsigDB v5). *P* < 0.05 was deemed significantly enriched.

### Single cell RNA sequencing (scRNA-seq)

mRNA expression for *AGO2* and differentially expressed miR-124-3p targets (AGO2 HITS-CLIP) across retinal cell types was retrieved from a publicly available scRNA-seq database deposited by Macosko et al. [54] (GSE63473, Gene Expression Omnibus). The cells in this database were obtained from whole retina cell suspensions of wild-type, dim-reared C57BL/6 mice. All data processing steps were performed with BioTuring Browser, version 2.7.48 for Mac OS (BioTuring Inc., San Diego, California, USA). Expression matrices were imported into BioTuring Browser in MTX format and quality-filtered for cells with min. 200 genes/cell and < 5% mitochondrial gene ratio, finally retaining 13,189 cells. Dimensionality reduction was performed by t-distributed stochastic neighbor embedding (t-SNE) on the first 30 principal components with perplexity set at 30. Next, graph-based clustering was performed (Louvain algorithm) and cell-types were annotated according to the cell markers supplied in the original publication. This dataset was queried for the expression of AGO2 and miR-124-3p targets and the results of this query were graphically displayed using ggplot2 package [55] in R [56].

### Western blotting

Retinas from dim and PD mice were lysed in CellLytic™ Cell Lysis Buffer (Sigma-Aldrich, MO, USA) supplemented with a protease inhibitor cocktail (Sigma-Aldrich). A total of 20 μg of protein was reduced and denatured then subjected to electrophoresis on a Novex™ 4–20% Tris-Glycine Mini Gel (Thermo Fisher Scientific). After separation, the protein was transferred to a nitrocellulose membrane (Bio-Rad, CA, USA), blocked in 3% BSA/PBS for 1 h, then incubated in primary anti-Argonaute-2 antibody (1:1000, ab32381, Abcam, Cambridge, UK) overnight at 4 °C. The following day, the membrane was incubated for 2 h in a HRP-conjugated Goat Anti-Rabbit IgG (H + L) secondary antibody (Bio-Rad) and developed with the ClarityTM Western ECL Substrate (Bio-Rad). After development, the membranes were imaged using the ChemiDoc™ MP Imaging System with Image Lab™ software (Bio-Rad). GAPDH (G9545, Sigma-Aldrich) was used a protein loading control.

### Immunohistochemistry

Immunohistochemistry was performed as previously published [14]. Briefly, mouse eyeballs were enucleated, fixed in 4% paraformaldehyde, dehydrated and embedded in Tissue-Tek® Optimal Cutting Temperature Compound (Sakura Finetek, California, USA). 12 μm cryo-sections were rehydrated, blocked for 1 h in 10% Normal Goat Serum then probed with primary antibody overnight at 4 °C. Immunofluorescence signal was developed with Alexa Fluor IgG secondary antibodies for 2 h at RT, followed by nuclei counterstaining with Hoechst 33342 (Sigma-Aldrich). Antibodies: anti-Argonaute-2, 1:500, ab32381, Abcam; anti-glutamine synthase, 1:500, Abcam; anti-rabbit IgG Alexa Fluor 488 nm and 647 nm, ThermoFisher.

### In situ hybridization

Complementary DNA was synthesised from retinal RNA as described previously [14]. A 445 bp region of the mouse *AGO2* mRNA (NM_153178.4) was amplified using the forward primer GAGACAGTCCACCTCTTGTGG and reverse primer GCCCAGAAGCAAACAACACC. The reverse primer was extended with a T7 RNA Polymerase tag ATATATTAATACGACTCACTATAGG at the 5′ end. A total of 35 PCR cycles (melting (95 °C, 15 s), annealing (60 °C, 15 s), extension (72 °C, 10s)) were carried out and the presence of the correct amplicon was verified using electrophoresis on a 1% agarose gel. After amplification, the PCR product was mixed with ¼ of ammonium acetate and 10 volumes of ice-cold absolute ethanol. The PCR product was centrifuged (13,000 g, 15 mins, 4 °C), then the pallet was washed with 70% ice-cold ethanol then re-precipitated (13,000 g, 2 mins, 4 °C). The ethanol was removed and the PCR product was reconstituted in Ultrapure water (Gibco, Thermo Fisher Scientific). The purity and concentration of the reconstituted PCR product was assessed on a ND-1000 spectrophotometer (Nanodrop Technologies, DE, USA). A riboprobe was transcribed from the PCR template using a T7 RNA polymerase (Promega, Madison, WI), incorporated into digoxigenin (DIG) SP6/T7 RNA polymerase (Roche, Basel, Switzerland), then hybridised to cryso-sections as previously published [57]. Optimum hybridisation for AGO2 probe was achieved at 59 °C overnight. Unbound probe was washed with saline sodium citrate (pH 7.4) at 60 °C and developed using nitro blue tetrazolium and 5-bromo-4-chloro-3-indolyl phosphate (NBT/BCIP) (Sigma Aldrich) for 60 min. The reaction was stopped by washing in ultrapure water and the slides were mounted with Aqua-Poly/Mount.

### Image acquisition and processing

Images were captured on a Nikon A1^+^ confocal microscope operated via NIS-Elements AR software (Nikon, Tokyo, Japan) acquiring sequential 0.3 μm z-stacks followed by maximum intensity projection processing. AGO2 in situ hybridisation labelling was captured either using a colour DS-Ri1-U3 colour camera fitted on the Nikon A1+ system or differential interference contrast (DIC) imaging. For DIC images, labelled areas were selected using 16-bit image thresholding in ImageJ v2.1 (NIH, Bethesda, MD, USA) [58] then pseudo coloured. For colocalization analysis, raw confocal files were imported into ImageJ v2.1 and converted to 16-bit. Consistent pixel intensity thresholds were applied to both channels using JACoP plugin [59] such that only area with clear labelling were included in the colocalization analysis then Pearson’s correlation coefficient was calculated. Colocalised pixel were pseudo-coloured using ColocalisationFinder plugin.

### Data availability and user-friendly exploration

Datasets supporting the conclusions of this article are available on the Sequence Read Archive (PRJNA606092) as part of the National Center for Biotechnology Information (NCBI). Processed RNA-seq data can be interactively explored at: https://genedatasets.shinyapps.io/ARMMI/

## Results

### Global miRNA and mRNA transcriptomes reveal key miRNA and inflammatory pathways involved in retinal degeneration induced by PD

**Fig. 1:**
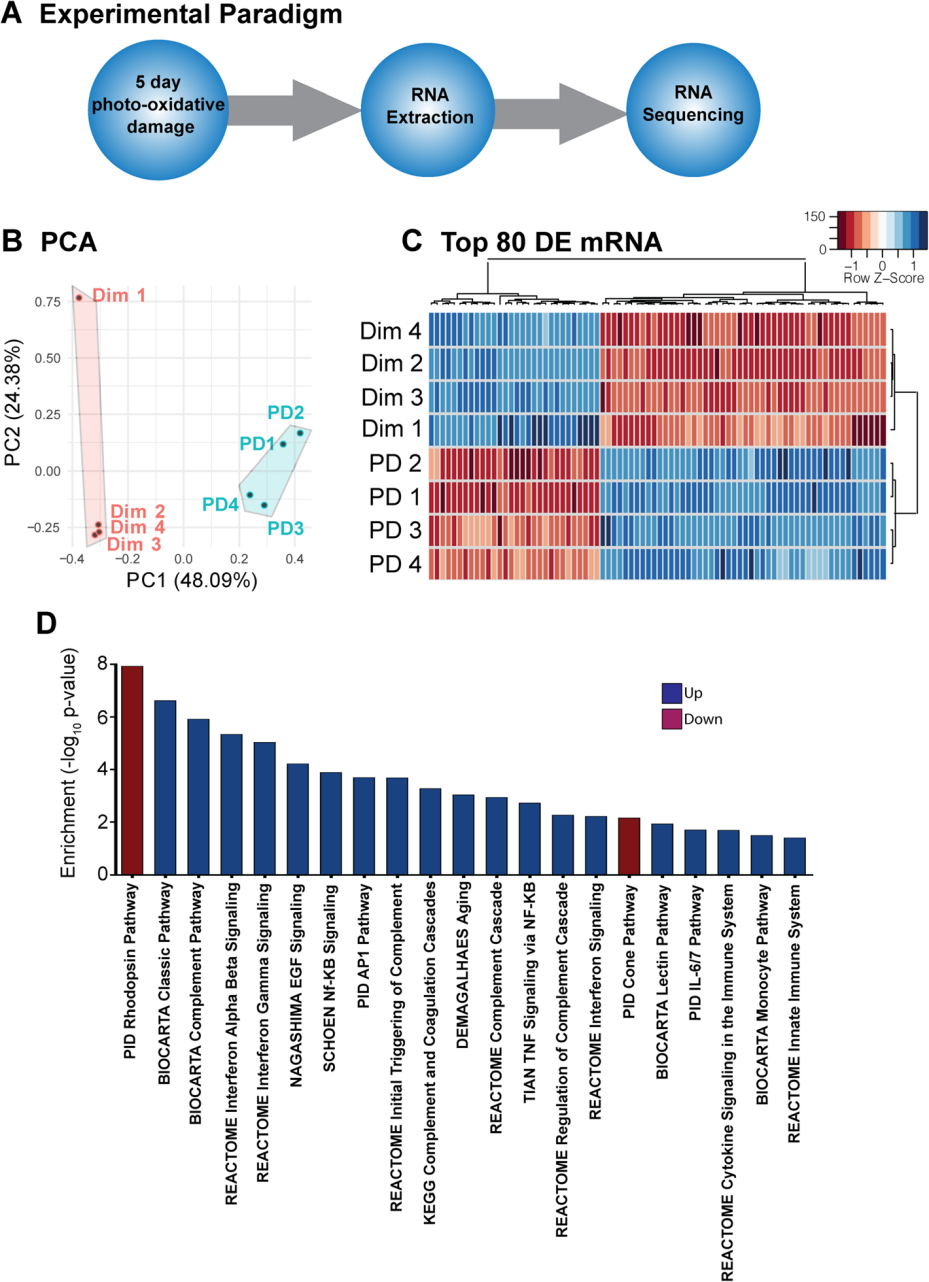
Global retinal mRNA expression profile determined by high-throughput sequencing (HTS). (**A**) Retinas from mice subjected to 5 days photo-oxidative damage (PD; *n* = 4), or dim-reared controls (dim; *n* = 4), were collected and RNA extracted. Sequencing libraries were prepared and subjected to HTS Illumina sequencing. Obtained reads were cleaned and trimmed and aligned to the mm10 mouse genome. (**B**) Principal Component Analysis (PCA) demonstrated tight clustering of the dim and PD groups with the exception of dim sample 1. (**C**) Hierarchical clustering was performed with for the top 80 differentially expressed mRNA and illustrated with a heat map. Sample specific expression level of each mRNA is indicated by the z-score color scale. (**D**) Gene set enrichment analysis (GSEA) for all differentially expressed mRNAs was performed against the C2 database with down-regulated pathways indicated in red and up-regulated pathways in blue (*P* < 0.05)

Retinal RNA was extracted and sequenced from dim and PD retinas to compare differences in gene expression following retinal degeneration (Fig. 1A). A two-dimensional principal component analysis (PCA) was employed to visualize differences in RNA profiles between the groups, which showed clear inter-group clustering with clear segregation between PD and dim retinas (Fig. 1B). Despite inherent animal variability displayed in the “dim 1” retina, the animals all responded similarly to PD with close intra-group clustering. Differential expression analysis identified 3018 mRNA transcripts to be significantly changed between dim and PD retinas based on an adjusted *P*-value of less than 0.05 (Fig. S1A)*.* Hierarchical clustering of the top 80 differentially expressed mRNA revealed strong correlations intra-group (Fig. 1C). Gene set enrichment analysis (GSEA) against the C2 collection from the Molecular Signatures Database (MSigDB) was used to decipher enriched pathways of these differentially expressed mRNAs. Enrichment analysis of the differentially expressed mRNAs revealed 203 significantly enriched terms/pathways (Fig. 1D, Table S5, *P* < 0.05). Among the most highly enriched terms were pathways involved primarily in the inflammatory response and innate immune system such as the monocyte pathway, cytokine and interleukin signaling, interferon signaling and many facets of the complement pathway including the lectin and classical pathways (Fig. 1D, Table S5) were all upregulated consistent with previous work demonstrating the key role of the innate immune response in the progression of retinal degenerations [17, 18]. Further, cone and rhodopsin pathways were downregulated (Fig. 1D, Table S5), indicating photoreceptor dysregulation consistent with the expected effects of the PD model [42].

**Fig. 2:**
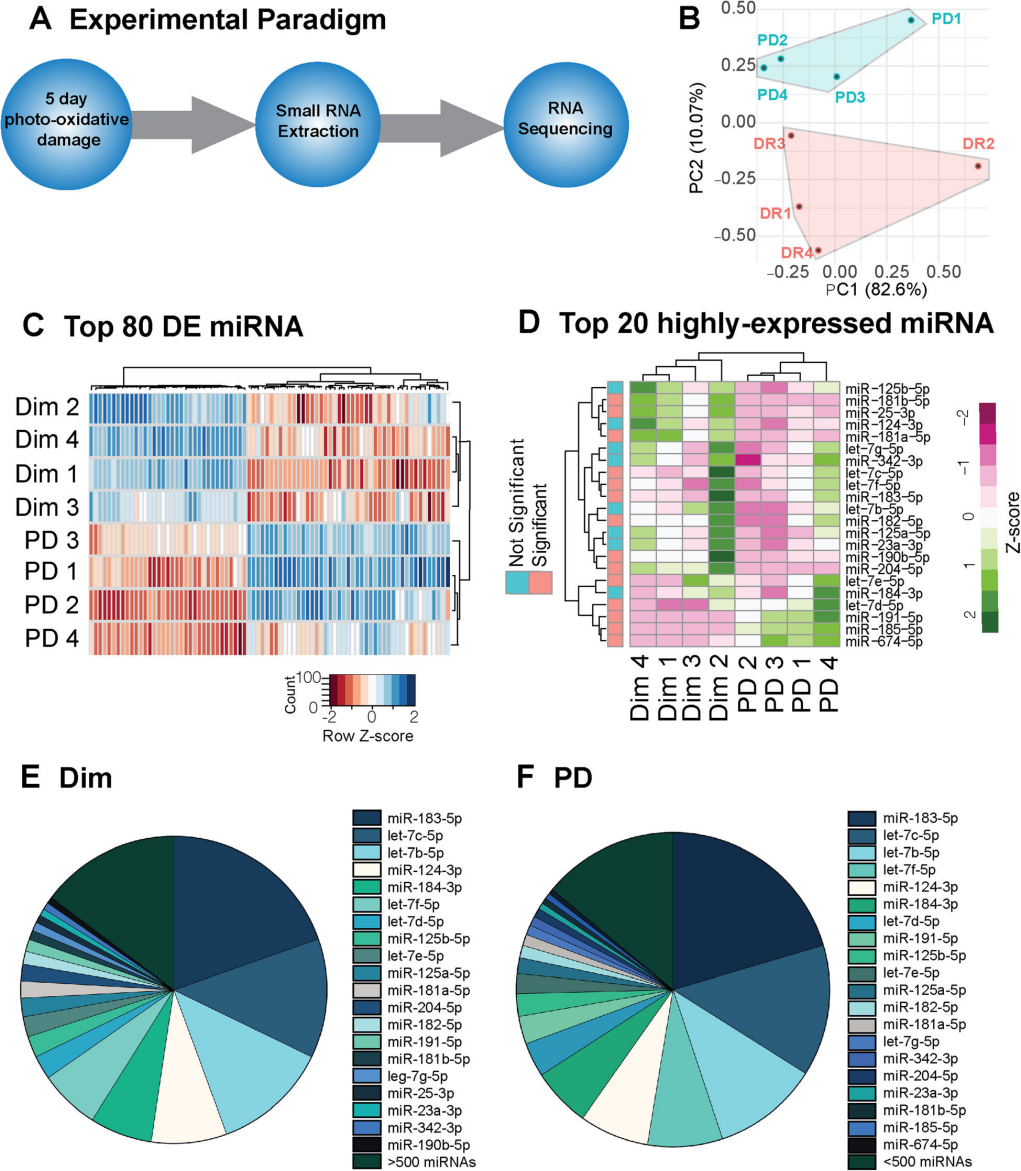
Global retinal miRNA expression profile determined by high-throughput sequencing (HTS). (**A**) Retinas from mice subjected to 5 days photo-oxidative damage (PD; *n* = 4), or dim-reared controls (dim; *n* = 4), were collected and small RNA extracted. Sequencing libraries were prepared and subjected to HTS Illumina sequencing. Obtained reads were cleaned and trimmed and aligned to mature mouse miRNA sequences (miRBase). (**B**) PCA analysis demonstrated good clustering of samples inter-group. (**C**) Hierarchical clustering was performed with for the top 80 differentially expressed miRNA and illustrated with a heat map. Sample specific expression level of each miRNA is indicated by the z-score color scale. (**D**) Hierarchical clustering was also performed for the top 20 most highly-expressed miRNAs with significant miRNA color coded on the left-hand panel (*P* < 0.05). (**E-F**) Pie charts illustrating the expression level of the global retinal miRNA in the dim (**E**) and PD (**F**) samples

Concurrent with the mRNA analysis, small RNA were extracted and sequenced to conduct analyze specifically analyze differences in miRNA profiles between dim and PD retinas. miRNA analysis identified 287 significantly differentially expressed miRNAs between dim and PD retinas (Fig. S1B, *P* < 0.05). PCA showed clear clustering between the two groups suggesting a unique miRNA profile and differences between dim and PD retinas (Fig. 2B). The top 80 differentially expressed miRNAs showed highly similar expression within the same group, but considerably different expression between dim and PD groups (Fig. 1C). Among the top 10 most significantly different miRNAs were those known to be involved in maintaining retinal neuronal homeostasis [60], including miR-9-5p (down-regulated), miR-211-5p (down-regulated) and miR-191-5p (up-regulated) (Fig. 1C, S1B). Taking the top 20 most highly-expressed miRNAs, there was again a distinct difference between dim and PD groups with 14 out of the 20 miRNAs statistically significant (*P* < 0.05, Fig. 1D). Interestingly, the top 20 most abundant miRNAs represented 85% of the total retinal miRnome in both the dim and PD (Fig. 1E-F) retinas, with let-7 family members, photoreceptor-enriched miR-183-5p and miR-182-5p as well as neuron-enriched miR-124-3p the most highly expressed miRNAs. Collectively, these results demonstrate that only a small set of miRNAs represents the overwhelming majority of the total retinal miRnome and that they are largely involved in pathways regulating the innate immune system.

### AGO2 HITS-CLIP reveals active retinal miRnome and mRNA targetome

**Fig. 3:**
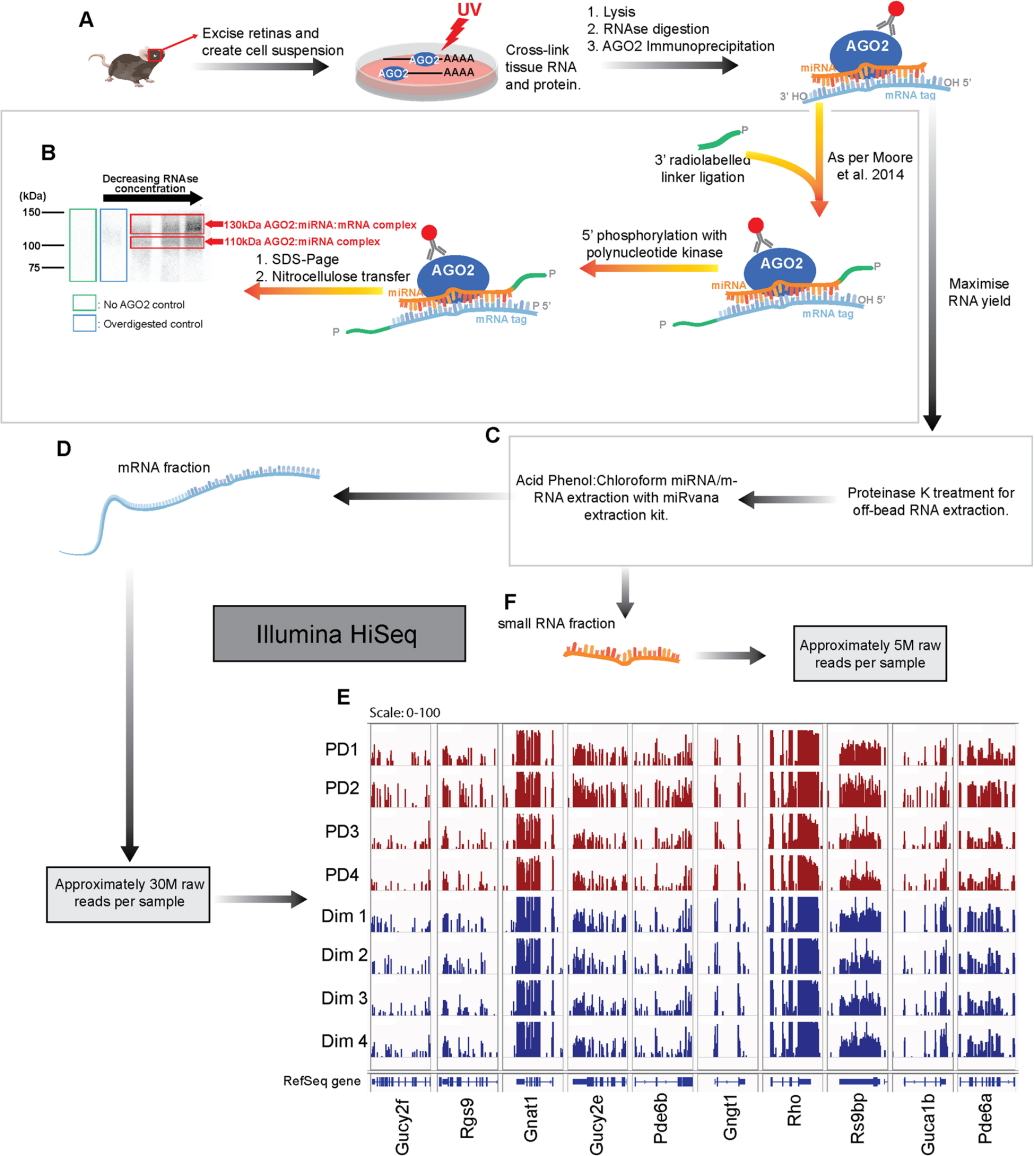
AGO2 HITS-CLIP workflow in mouse retina. (**A**) Mice were subjected to 5 days of photo-oxidative damage, dim-reared mice were used as control. Retinas were excised from eyeball and diced into a fine suspension, which was UV cross-linked. Tissue was lysed, digested with RNase and AGO2-containing complexes immunoprecipitated as described previously (Moore et al. 2014). (**B**) RNA of purified complexes was radiolabeled by 3′ linker ligation as previously described (Moore et al. 2014). Complexes were separated by SDS-PAGE, transferred to membrane and exposed to film. Autoradiogram showing 110 kDa and 130 kDa complexes representing the AGO2:miRNA and AGO2:miRNA:mRNA complexes, respectively. No AGO2 antibody (green box) and RNase over-digested (blue box) controls were included. (**C**) To maximise yield, RNA was extracted from complexes by on-bead proteinase K treatment. Total RNA was purified by phenol/chloroform extraction and the supernatant further purified using the *mir*Vana Kit to separate (**D**) mRNA. (**E**) Positional read coverage of key genes within the rhodopsin pathway shows considerable homogeneity between intra-group samples. (**F**) Small RNA was isolated for miRNA analysis

Whilst the global transcriptome analysis provides a snapshot of the molecular and physiological processes that occur in response to photoreceptor retinal degeneration, AGO2 HITS-CLIP was performed to decipher the functional miRnome and its respective targets (Fig. 3A-C).

mRNA sequencing yielded ~ 30 million raw reads per sample, which were cleaned and aligned to the mm10 mouse genome (Fig. 3D, Table S3). Data consistency and reproducibility was visually evident amongst the groups looking at the positional read coverage of key visual transduction genes (Fig. 3E). Small RNA sequencing yielded ~ 5 million raw reads per sample, which were trimmed and aligned to the mature mouse miRNA sequences obtained from miRBase (Fig. 3F, Table S4). Differential analysis did not reveal any change in accumulation of AGO2-bound miRNAs between dim and PD samples. This demonstrates the importance of functional validation in miRNA analysis as the non-AGO2 HITS-CLIP results differed showing a change in expression. To understand the dynamics within both homeostatic and degenerative conditions, we continued our analysis into dim and PD datasets separately.

**Fig. 4:**
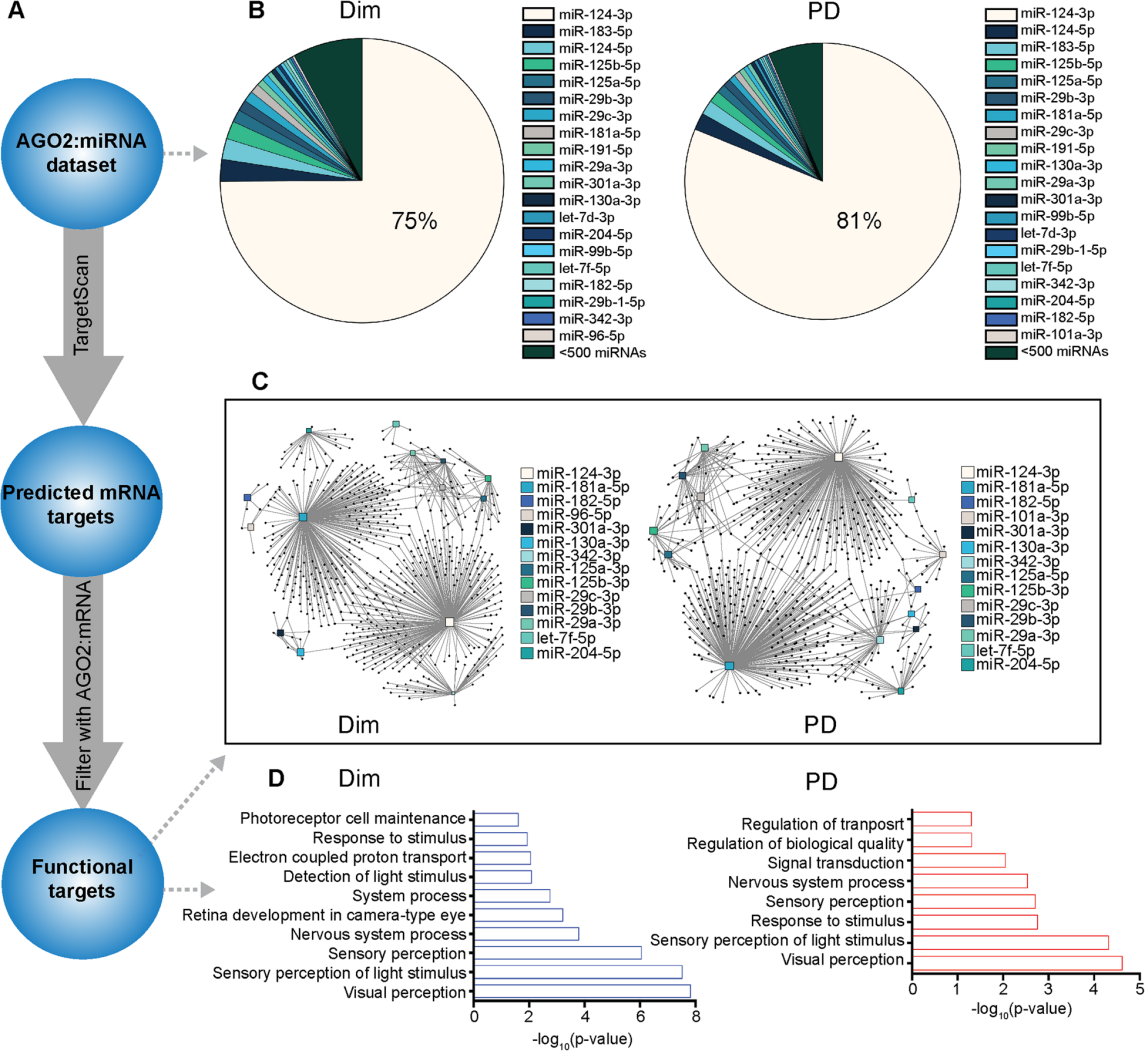
Network analysis of the top 20 DR and PD retinal AGO2-bound miRNAs. (**A**) Schematic of the analysis conducted for the top 20 DR and PD AGO2-bound miRNAs. (**B**) Pie chart showing the AGO2-bound miRnome of DR and PD retinas (see also Table S3A). The top 20 miRNAs are individually listed, with miR-124-3p comprising approximately 75 and 81% of the miRnome respectively. (**C**) Target network analysis for the top 20 AGO2-bound miRNAs was performed with miRNet using all AGO2-bound mRNAs as putative target set (see also Table S3B). The results for 14 miRNA are shown, the remaining six miRNAs were not annotated in the system. Each miRNA is represented by a square box, with its size indicating the number of interactions. Black dots represent interacting mRNAs connected by lines to the respective miRNA. (**D**) Gene ontology (GO) analysis was performed using the most abundant AGO2-bound mRNA transcripts identified in DR and PD retinas to view the most enriched biological processes involved (*P* < 0.05)

From the identified miRNA in the AGO2-bound miRNA dataset, the top 20 miRNA were run through TargetScan to get an output of their predicted targets (Fig. 4A). The predicted targets were then filtered through our AGO2-bound mRNA dataset to identify functional targets in the retina (Fig. 4A). 556 AGO2-bound miRNAs in dim retinas were identified. Interestingly, miR-124-3p was the most prominent, representing 75% of the AGO2-bound miRnome (Fig. 4B). Photoreceptor specific miR-183-5p (part of a cluster miR182/96/183) [1, 61–64] was the second most abundant miRNA followed by miR-124-5p. The remaining top 10 miRNAs included miR-125b-5p, miR-125a-5p, miR-181a-5p, miR-191-5p and three members of the miR-29 family: miR-29a-3p; miR-29b-3p; and miR-29c-3p. The other two members of the photoreceptor cluster, miR-182-5p and miR-96-5p, were among the top 20 AGO2-bound miRNAs in dim retinas. miR-301a-3p, miR-130a-3p, miR-204-5p, miR-99b-5p, miR-29b-1-5p, miR-342-3p and two members of the let-7 family (let-7d-3p and let-7f-5p) completed the top 20. 554 AGO2-bound miRNAs were identified in PD retinas (Fig. 4B). Similar to the dim retinas, miR-124-3p was the most abundant constituting 81% of the AGO2-bound miRnome. miR-124-5p and miR-183-5p were second and third most common miRNA bound to AGO2, respectively. The top 20 AGO2-bound miRNA identified in PD retinas were almost identical to those found in dim retinas, with each having slightly different overall abundances (Fig. 4B).

To determine the size and breadth of the predicted miRNA/mRNA target networks, miRNet analysis was performed with the top 20 AGO2-bound miRNAs using all identified AGO2-bound mRNAs as the putative target set. This revealed mRNA targets for 14 miRNAs (Fig. 4C) with the remaining six (miR-183-5p, miR-124-5p, miR-191-5p, let-7d-5p, miR-99b-5p, miR-29b-1-5p) not well annotated in miRNet. Among them miR-124-3p and miR-181-5p returned the majority of mRNA target interactions, with 223 and 233 interactions each, respectively (Fig. 4C). The PD dataset also yielded networks for 14 of the top 20 AGO2-bound miRNA (Fig. 4C). As with the dim retinas, miR-124-3p and miR-181-5p showed the highest number of interactions (Fig. 4C). The most enriched biological processes for the AGO2-bound mRNA ranked based on abundance were somewhat similar in both dim and PD datasets with processes involved in visual processing, such as visual perception and sensory perception of light stimulus, being the most enriched in both (Fig. 4D).

Here, breadth of the miR-124-3p networks and size of their population amongst the functional miRnome of both dim and PD retinas was demonstrated. Further, the functional AGO2-bound miRnome is overwhelmingly involved in standard visual processes of the retina, indicating a homeostatic role for vision and retinal maintenance that remains important under stress.

### Retinal AGO2-bound miRNAs shift their mRNA targetome following PD

**Fig. 5:**
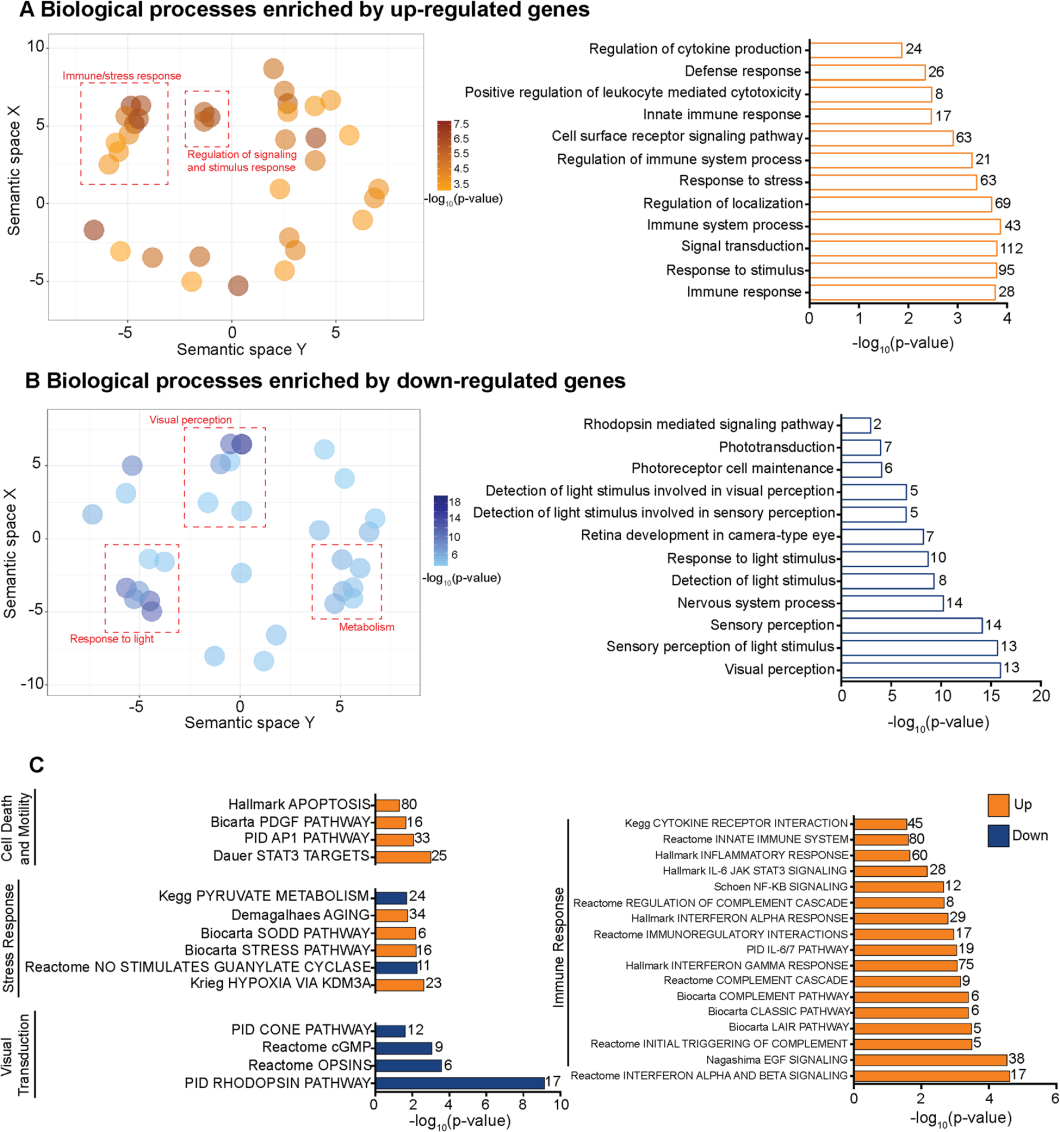
Pathway enrichment of differentially bound AGO2 mRNA in PD retinas. (**A**) Up-regulated AGO2-bound mRNA were analysed for GO biological process enrichment with significant terms clustered with REVIGO for visual analysis. (**B**) The same process was performed for down-regulated genes in PD retinas. (**C**) Gene set enrichment analysis (GSEA) was performed using a ranked list of the differentially AGO2-bound mRNAs against the C2 database for enriched pathways. Selected pathways were separated into four groups: cell death and motility; stress response visual transduction; and immune response (*P* < 0.05). Numbers accompanying the bars represent the number of genes associated with the respective pathway

Although AGO2-bound mRNA enriched GO terms between dim and PD samples were similar, differences in mRNAs contributing to those GO terms was investigated. Differential expression analysis was performed and identified 96 mRNAs that are differentially bound to AGO2 following PD with 44 up-regulated and 52 down-regulated (*P* < 0.05). Functional enrichment analysis for gene ontology biological processes on the AGO2-bound mRNA dataset ranked by *p*-value was then conducted. The datasets were split into two groups: 1) up-regulated genes in PD compared to dim (Table S6); 2) down-regulated genes in PD compared to dim (Table S7). Enriched biological processes were clustered using REVIGO to better visualize the gene ontology output. For up-regulated genes there were obvious clusters for the immune response and regulation of signaling and response processes (Fig. 5A). The majority of enriched terms, as measured by log_10_p-value, were immune and stress response related. For down-regulated genes there was clustering observed for visual perception, metabolism and light response processes (Fig. 5B). A breakdown of the enriched GO processes corroborates the clusters with the majority of the GO terms associated with the visual pathway. Full GO including molecular function and cellular component can be found in Tables S8 and S9 in the supplementary data.

To further explore the specific pathways up- or down-regulated in the AGO2-bound mRNA dataset, gene set enrichment analysis (GSEA) against the C2 collection from the MSigDB was performed [65, 66]. Selected pathways were then sorted into the following categories: *cell death and motility; stress response; visual transduction;* and *immune response* (Fig. 5C). Enriched pathways under the *cell death and motility*, *stress response* and *immune response* categories were largely up-regulated in PD retinas. Included in these categories are the complement pathway, NF-KB signaling, IFN alpha and beta signaling and apoptotic pathways, all known to play roles in retinal degeneration [18]. Notably, the four pathways categorized under *visual transduction* all were down-regulated. These results indicate that, whilst maintenance of the visual system remains a priority for the retinal miRnome, there is a nuanced change in mRNA targeting by active AGO2-bound miRNAs following photo-oxidative damage. The activity of AGO2-bound miRNAs appears to shift its focus from visual transduction, to the retinal response under duress particularly targeting genes involved in inflammatory pathways and the immune response.

### miR-124-3p undergoes shift in seed binding region following PD

**Fig. 6:**
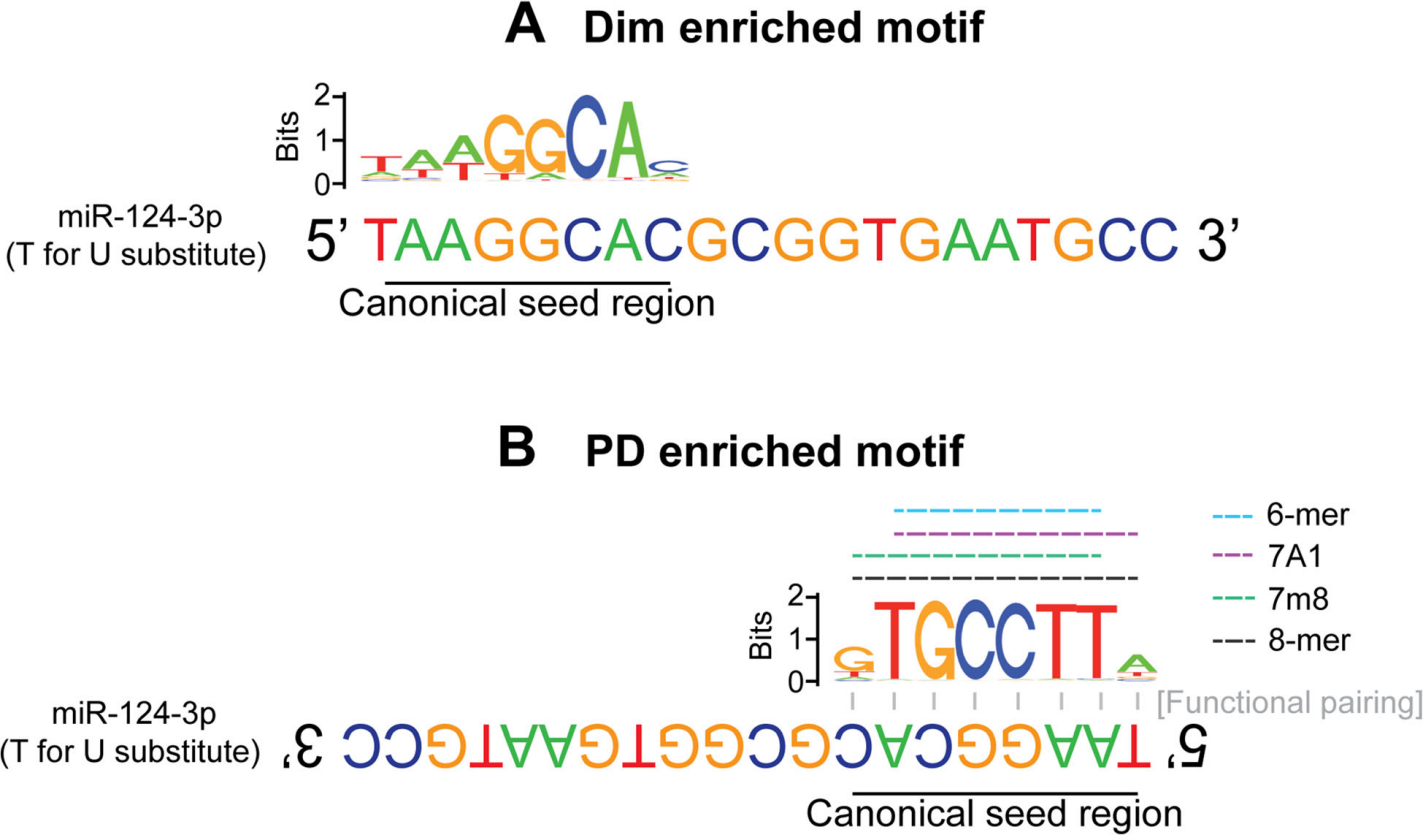
Enriched sequence motifs of AGO2-bound mRNA. Peak calling (HOMER) of reads mapped to AGO2-bound mRNAs was performed for DR and PR samples. Peaks were annotated according to transcript region and subjected to motif enrichment analysis (HOMER). The most highly enriched motif for each sample is shown here. For easy orientation, the cDNA sequence of miR-124-3p is displayed at the top, with positions 1–6 indicated by the dashed box and positions 2–8 indicated by the solid box. (**A**) The top most enriched binding motifs of AGO2-bound mRNAs in dim retinas was the exact sequence of the miR-124-3p seed region. (**B**) The most enriched binding motifs of AGO2-bound mRNAs in PD retinas are the reverse complement of positions 2–8 (or 1–8 for the 8-mer motifs) of the miR-124-3p binding regions encompassing all canonical seed regions as indicated with the colored lines

Given the changes in AGO2-bound mRNAs following PD, the binding interactions of the AGO2-bound miRNAs with their targetome was analyzed next. We performed peak calling of the reads aligning to the 3531 unique AGO2-bound mRNA transcripts identified in dim and PD retinas. Identified peaks were annotated according to transcript region (according to RefSeq) and subjected to motif enrichment analysis (HOMER). The most enriched sequence motifs within the 3′ UTRs of the AGO2-bound mRNAs in dim retinas matched the exact sequence of the miR-124-3p seed region (Fig. 6A). Where as in PD retinas we found that the most enriched sequenced motif matched the eight nucleotide reverse complementary seed region of miR-124-3p indicating regulation of miR-124-3p specific targets. All four canonical seed regions were represented: 6-mer (reverse complement of miRNA positions 2–7); 7A1 (reverse complement of miRNA positions 2–7 with an adenine base); 7m8 (reverse complement of miRNA positions 2–8); and 8-mer (reverse complement of miRNA positions 2–8 with an adenine base) (Fig. 6B). To determine the extent to which miR-124-3p might regulate its targets within the retina, AGO2-bound mRNAs that are predicted binding partners of miR-124-3p as indicated by TargetScan were tabulated (Table S10). Up-regulated targets, as represented by a positive log fold change, in PD retinas compared to dim is indicative of increased binding. A number of these transcripts have well-established links to retinal degeneration, including chemokine C-C motif ligand 2 (*Ccl2*) [67] and rho-associated protein kinase 2 (*Rock2*) [68]. The cellular location and baseline expression of the top 10 most differentially expressed miR-124-3p targets are highlighted in Fig. 6B using publicly available single-cell RNA data as described in the next section.

Taken together, this data indicates that following PD, a change in miR-124-3p miRNA target activity occurs, which coincides with an increased AGO2 movement within the retina. These changes may reflect a key aspect of miRNA function under duress that results in a translocation within retinal maintenance cells to regulate differentially expressed disease-specific pathways in the retina.

#### Expression of Ago2 changes upon retinal damage

**Fig. 7:**
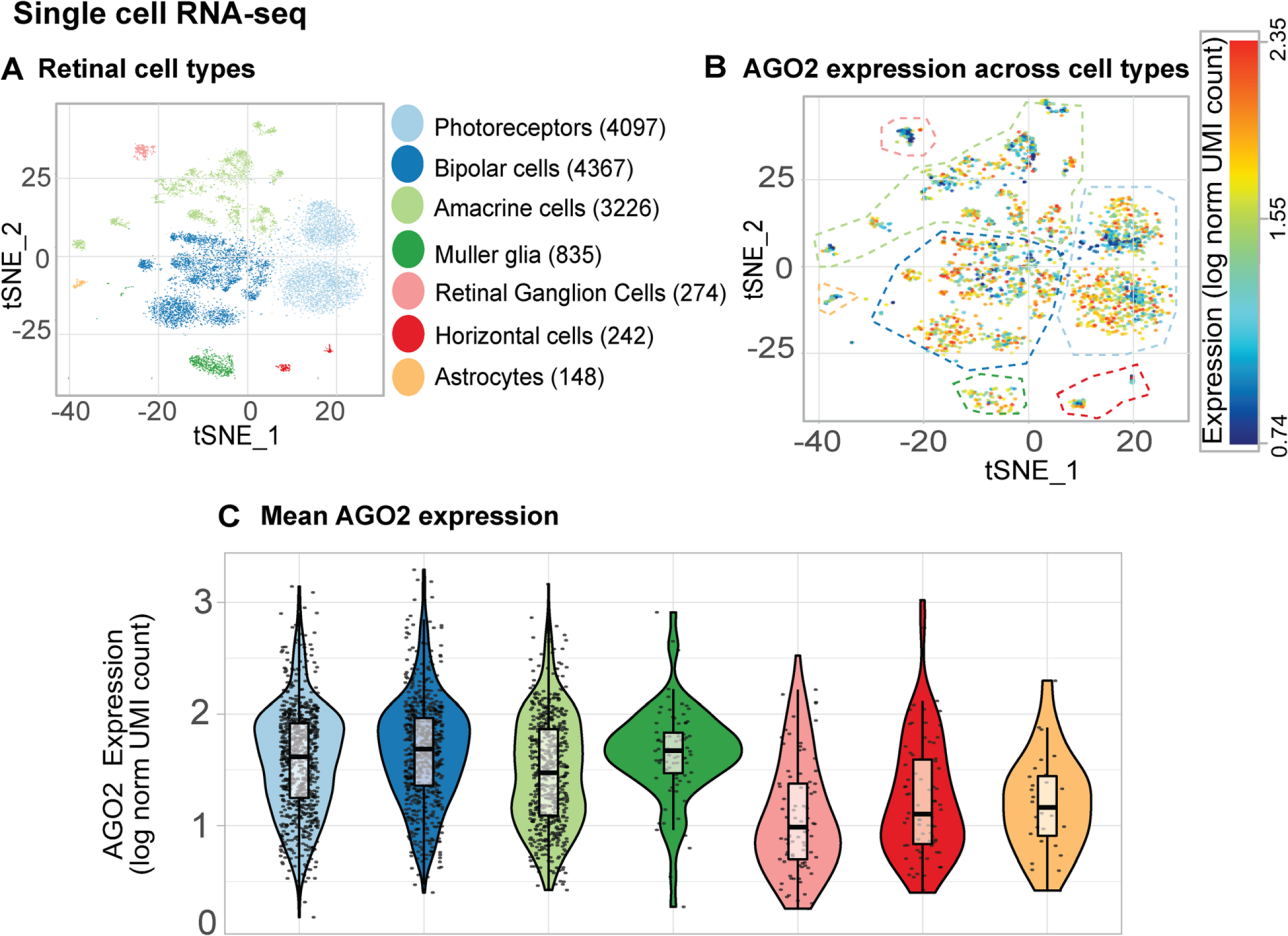
Cellular location and expression of AGO2 mRNA and mir-124-3p targets derived from publicly available scRNA-seq data. (**A**) t-SNE plot showing 19 retinal cell populations ascribed to a major class of retinal cell types indicated by colour-coding. Value between parentheses show the number of cells within each cluster. Colour coding applies to all subsequent plots in this Fig. (**B**) *AGO2* expression levels across all cells with AGO2 expression > 0. Areas enclosed by dashed lines indicate the predominant cell population occupying that region of the t-SNE plot identified according to the colour-coding depicted in A. (**C**) AGO2 expression for each cell class depicted in ii represented as box plots showing the median values, Q1 (25th) and Q3 (75th^)^ quartiles. Whiskers extend 1.5 times above and below and interquartile range. Each point corresponds to the expression of a single cell within that cluster

**Fig. 8:**
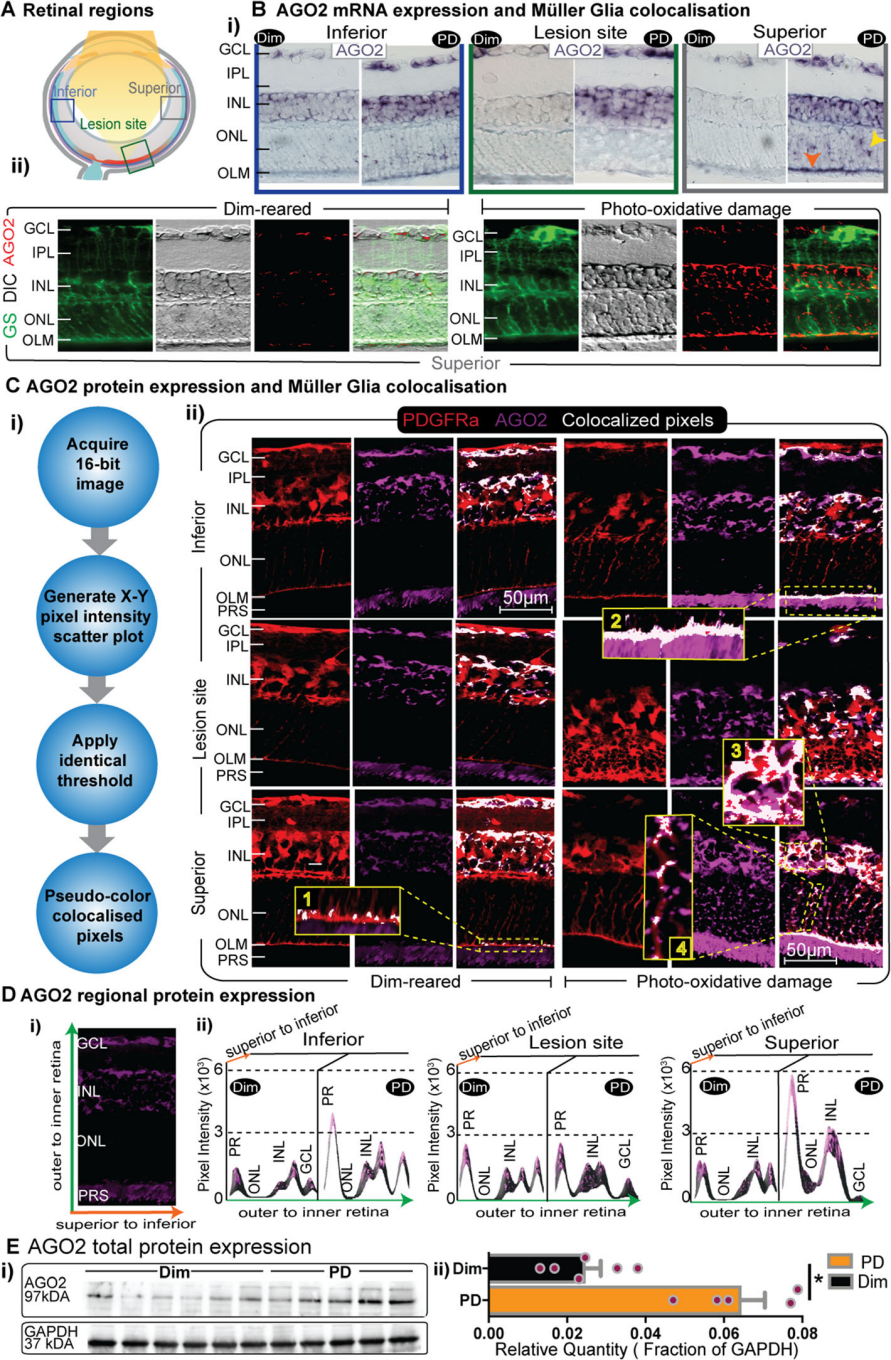
AGO2 gene and protein expression changes following PD. (**A**) Diagram indicating retinal regions where histological measures were taken. Lesion site refers to the area most damaged by PD. (**B**) (i) AGO2 in situ hybridization in dim and PD retinas at the three retinal sites indicated in A. AGO2 mRNA increased at all three locations and is distributed across the GCL, INL in the inferior retina and lesion site. In the superior retina, AGO2 mRNA expression is also visible within the ONL (yellow arrow) and the OLM (orange arrow). (ii) Co-labelling of AGO2 mRNA and glutamine synthetase in the superior retina showing colocalization along the OLM and Müller glia processes. Gray micrographs are DIC images of AGO2 in situ hybridization (as shown in i). Red labelling are pseudo-coloured pixels corresponding to the area positive for AGO2 labelling (dark spots in the adjacent DIC image). (**C**) (i) Image analysis pipeline used for colocalization analysis. (ii) Immunohistochemistry showed AGO2 protein expression is confined within the GCL, INL and photoreceptor segments in dim retinas. Following PD, AGO2 protein expression is elevated within the INL and photoreceptor segments. Co-localization of AGO2 labelling with Müller glia visualized using a reporter mouse strain showed distinct colocalization along the inferior and superior OLM (inset 2), Müller glia processes (inset 4) and Müller glia cell bodies (inset 3). In the absence of PD limited colocalization is seen in the superior retina (inset 1). (**D**) (i) Axis description for plots shown in ii. (ii) AGO2 protein expression profile quantified across retinal layers and depicted as pixel intensity (y-axis), along the outer to inner retinal direction (green x-axis) and superior to inferior (orange z-axis). (**E**) (i) AGO2 western blot and corresponding GAPDH loading control. (ii) GAPDH-normalized densitometry values for AGO2 (Error bars show SEM, * *P* < 0.05, Student’s t-test)

AGO2 HITS-CLIP was performed using whole retinal lysates, however the expression of AGO2 across retinal cell types remained to investigated. The re-analysis of scRNA-seq data [54] indicated that AGO2 mRNA is present in both retinal neurons (photoreceptors, bipolar cells, amacrine cells, horizontal cells and retinal ganglion cells) and retinal glia (Müller glia and astrocytes; Fig. 7A and B) under normal dim conditions. Within each cluster AGO2 expression was variable ranging from 0.74 to 2.35 log normalized UMI counts (Fig. 7B) with average expression of similar magnitudes for photoreceptors, bipolar cells, amacrine cells and Müller glia and comparatively reduced expression for retinal ganglion cells, horizontal cells and astrocytes (Fig. 7C). To further study the expression of AGO2 within the mouse retina, we probed both AGO2 mRNA and protein expression using immunohistochemistry, western blot and in situ hybridization at a range of retinal eccentricities in dim and PD retinas (Fig. 8A).

In line with the scRNA-seq, in situ hybridization showed *AGO2* mRNA expression in heathy retinas within the cell bodies of RGCs, the inner and outer regions of the INL with an accentuation in expression in the peripheral retina (cf central retina) (Fig. 8B i). PD resulted in increased *AGO2* mRNA expression, localized within the INL and GCL in the inferior retina and at the lesion site (Fig. 8B ii). The superior retina proximal to the lesion site also showed increased AGO2 expression across the INL and GCL, however AGO2 was also detected within the ONL here and the outer limiting membrane (OLM) formed by the outer extremities of the Müller glia processes (Fig. 8B ii). To verify that the AGO2 mRNA was indeed expressed in the OLM, co-labelling with glutamine synthase (Müller glia marker) was performed showing distinct *AGO2*-Müller glia colocalization within the OLM and further revealing AGO2 mRNA expression along the processes of Müller glia traversing the ONL (Fig. 8B ii).

Immunohistochemistry for AGO2 corroborated the changes observed at gene level, primarily showing that AGO2 is expressed in the GCL and INL where its expression increases following photo-oxidative damage (Fig. 8C ii). In addition, AGO2 protein was expressed in the photoreceptor segments and the PD dependent increase here was evident in both the superior and inferior retina, but not at the lesion site (likely due to a focal loss of photoreceptor segments here) (Fig. 8C ii). Co-labelling of AGO2 protein and Müller glia (using a Müller glia reporter mouse strain) showed distinct colocalization within the OLM, along the Müller glia processes traversing the OLM, and a more pronounced localization with the cell bodies of Müller glia in the INL in the superior retina compared to the other retinal sites examined (Fig. 8C ii). AGO expression changes across the retina were also depicted graphically highlighting the changes in the photoreceptor segments and within the central region of the INL (Fig. 8D i and ii). Finally, changes in bulk AGO2 protein expression were measured by western blot indicating that an increase of approximately 3-fold occurs following PD (*P* < 0.05, Fig. 8E i and ii).

Taken together, these data indicate that AGO2 has a pan-retinal expression primarily within the nuclear layers of the retina and the photoreceptor segments. Photo-oxidative damage induces a dynamic increase both AGO2 gene and protein expression and some fraction of this increase occurs in Müller glia, most prominently in at the OLM.

## Discussion

miRNAs are the most studied non-coding RNA in the central nervous system (CNS) [69] including the retina [60]. Despite this, the specific miRNAs required to maintain retinal homeostasis or to respond to retinal degeneration have not been identified or well-studied. Studies characterizing the retinal miRnome under homeostatic conditions have utilized small RNA sequencing technology to investigate the total retinal miRNA expression profile with high resolution and sensitivity [70]. We built on these previous findings by performing HTS of the global retinal mRNA and miRNA in both the healthy retina and degenerating retina. We found that both global miRNA and mRNA expression profiles are highly regulated in response to PD. Further, we showed that several inflammatory-related pathways are highly enriched and highlight key mRNAs integral to these pathways. We extended this study by utilizing AGO2 HITS-CLIP, to allow the in vivo identification of the functionally active AGO2-bound mRNAs and miRNAs. This enabled us to characterize the functional retinal miRnome. We observed no change in AGO2-bound miRNAs in response to PD, but interestingly observed a change in the AGO2-bound mRNA targets suggesting a shift in the action site of the miRNA silencing machinery. This mechanism likely underpins the role miR-124-3p plays in retinal degeneration as we have previously shown translocation of miR-124-3p through extracellular vesicles (EV) [14, 71] between retinal cell layers where miR-124-3p may modulate a new subset of distinct targets. Further analysis using motif enrichment of AGO2-bound mRNAs revealed a change in the seed region binding activity of the highly expressed miR-124-3p following PD. Analysis of AGO2 location showed expression in distinct cell types with histological staining demonstrating that AGO2 mRNA and protein accumulation appears to be expressed within Müller glia – a likely destination for miR-124-3p following the degeneration-dependent translocation of miR-124-3p as we have previously reported [14]. Moreover, the AGO2-bound miRNAs and mRNAs were linked to key pathways already identified as regulators of human AMD pathogenesis, such as the complement cascade, immune response and cell death [17, 18]. Taken together, these data demonstrate a dynamic interaction of the retinal AGO2 miRnome with its targetome which is influenced by the tissue environment. Therefore, our data provide important new insight into miRNA functionality during neurodegeneration, and identification of multiple miRNA-mRNA interactions of biological importance in retinal degenerations.

### miRNA expression is altered as a consequence of photoreceptor specific retinal degeneration

Previous reports have suggested that the vast majority of cellular miRnomes are comprised of only a few highly abundant miRNAs [72–75]. We used HTS to provide a snapshot of the retinal mRNA and miRNA composition and revealed that approximately 85% of the retinal miRnome consists of only 20 different miRNAs in both the healthy and degenerating retina. This includes key members of the photoreceptor cluster miR-183/96/182, which are involved in the development and homeostasis of photoreceptor cells [76] and miR-124-3p, a neuronally-enriched miRNA that plays a role in neuron homeostasis [31–34]. This data suggests that regulation of neuronal homeostasis plays an overarching role not only in the healthy, but also the degenerating retina. It is therefore not surprising that the dim and PD AGO2-bound miRnomes majorly comprised miRNAs that are known to play a role in retinal development and homeostasis, such as the photoreceptor cluster miR-183/96/182, miR-204-5p, let-7a-5p and miR-124-3p. In fact, we have previously characterized miR-124-3p, and shown that supplementation of miR-124-3p during retinal damage preserves retinal function, protects against photoreceptor death and reduces inflammation [14]. miRNet analysis showed that AGO2-bound miR-124-3p and miR-181a-5p (another highly-enriched retinal miRNA [77]) displayed extensive AGO2-bound mRNA target networks, further strengthening the notion that only a few highly enriched miRNAs are responsible for the majority of regulation of a dynamic group of mRNA targets [78–83]. However, despite significant changes in global miRNA expression in the damaged retina, we observed no significant change to the AGO2-bound miRnome. It is possible that the globally altered miRnome reflects a miRNA transcription and processing state that is either are not functional, e.g. those miRNAs are not incorporated into AGO2, or are operating via a non-canonical miRNA pathway [84, 85]. Further studies in this area are required to explore the non-canonical miRNA pathways in retinal degenerations.

### miRNA are important for maintaining retinal homeostasis and regulating innate immunity

GO term and pathway analyses of the global mRNA HTS showed that photoreceptor maintenance and visual cycle were highly enriched in both healthy control and degenerating retina. This was also true for the AGO2-bound mRNAs in both dim and PD, again indicating that maintenance of the visual system remains a regulatory priority in the retina. However, the global mRNA HTS also revealed that many of the pathways enriched in PD are involved in retinal inflammation [18]. Similarly, GO term and pathway analysis of the differentially AGO2-bound mRNAs showed significant enrichment of pathways of the immune response, regulation of defense, regulation of immune system processes and response to stress. Further, we demonstrated that there was a significant association with visual processes when it came to down-regulated targets. Up-regulated targets were involved in the inflammatory and stress responses. These findings are not unexpected, as these pathways have previously been strongly implicated to play a role in the progression of retinal degeneration [42, 86–89]. However, what we have demonstrated here is the influence that miRNA have on retinal inflammation in addition to dampening their influence on visual transduction in a stressed environment.

The immune response, particularly the innate immune response, is well-established to influence the progression of retinal degenerations (reviewed in [18]). Pathways involved in the innate system such as the interleukin and cytokine release were also significantly enriched in our differentially expressed dataset. Another highly represented pathway in our dataset was the complement system, which has been previously identified as a major instigator of inflammatory dysregulation in AMD [86–89]. Given this high immune pathway representation in this data, determining the miRNA binding sites within these miRNAs may lead to advances in potential therapeutic options targeting key inflammatory components involved in the degenerating retina.

### miRNA binding sites change in response to retinal degenerations

To understand the change in miRNA binding sites in response to retinal degenerations, we used sequence motif enrichment analysis within the 3′ UTRs of AGO2-bound mRNAs identified by HITS-CLIP. We demonstrated that the most enriched motif in PD were the reverse complementary canonical seed sequences of miR-124-3p indicative of high miR-124-3p binding and activity in the retina during degeneration. Interestingly, dim retinas showed a different enriched motif and with the actual seed sequence of miR-124-3p determined to be the most enriched. While understanding the biological relevance of this interaction is outside the scope of this work, we suggest that it may point to a self-regulatory system whereby miR-124-3p is being bound to itself under homeostatic conditions by its passenger strand. During the process of miRNA maturation, miRNA are loaded onto AGO2 in their duplex form before being unwound to generate a mature RISC [90]. We have previously shown miR-124-3p to be largely concentrated along the OLM in healthy normal retina, before translocating to the inner retina following photoreceptor specific retinal damage [14, 71]. It may be possible that miR-124-3p sits in a reservoir as a miRNA duplex and is more preferentially unwound under duress [91]. An alternative hypothesis is that miR-124-3p is present naturally where it is produced and requires an upregulation of AGO2 to be unwound. Extensive research is needed to uncover the complexities of miR-124-3p biogenesis and function and its potential alteration in activity during health and disease.

It is apparent, however, that there is a change in binding behavior of active miRNA following retinal damage with miR-124-3p activity enriched during PD. We postulate that miRNA activity in the damaged retina is different compared to the homeostatic state as our data demonstrates that the miRNA targets differ to regulate more inflammatory processes when the retina is under duress. It is likely that retinal degenerations cause existing miRNA populations to regulate different targets rather than transcribe novel miRNAs, which has been suggested in previous publications [92]. This study is the first indication that such a regulatory system also operates in the retina. The alternative regulation of target mRNAs is likely due to a movement of miRNA activity within the retina in response to degeneration [14, 71, 93]. As aforementioned, our previous studies have shown miR-124 to move to the Müller glia to regulate the expression of CCL2 upon damage onset [14]. We have demonstrated that this movement is, in part, mediated by EV, where miR-124 expression remains stagnant in the retina following EV biogenesis inhibition [14, 71]. This potential movement is further corroborated by the alternative labelling of AGO2 we have demonstrated here. AGO2 appears to concentrate along the Müller glial end feet in the OLM of the inferior and peripheral regions of the retina where there is less tissue degeneration from photo-oxidative damage. However, at the damage site, AGO2 expression seems to be more heavily expressed within the INL, including within the Müller glia bodies. Taken together, this may be indicative of a tightly balanced glia-to-neuron transfer of information as a means to combat against neurodegenerative insults and maintain homeostasis in the retina [14, 93, 94].

An alternative explanation to these observed differences could be that canonical miRNA biogenesis might not be the only pathway contributing to retinal homeostasis and retinal degenerations. Work in the field is beginning to shift focus towards miRNA binding events that occur outside of the 3′ UTR [95]. This might explain the difference in gene expression between global HTS and HITS-CLIP. Further analysis of the enriched peaks in our data may reveal binding sites in the coding region or the 5′ UTR. It is further possible that miRNA binding to its target is regulated by wobble or bulge nucleotides [96–98] or RNA modifications such as 6-methyladenosine, 5-methylcytosine or pseudouridine. Such alternative regulation could be indicative of non-canonical miRNA biogenesis in retinal degenerations and requires further exploration.

## Conclusions

In conclusion, our data suggests that miRNA are actively involved in regulating key genes and biological pathways involved in retinal degeneration. Whilst the AGO2-bound miRNA accumulation did not change significantly between healthy and damaged retinas, we demonstrate that there is a dynamic change in the miRNA target landscape mediated by favoring an extended seed sequence. Further, our data provide support to the notion that a change in miRNA targeting under stress is the result of miRNA movement throughout the retinal layers. Additional research into this mechanism will help uncover the intricacies of miRNA biology in neurodegenerative disorders.

## Supplementary Information

**Additional file 1 Fig. S1.** Volcano plots of the differentially expressed mRNA (**A**) and miRNA (**B**) in the global retina dataset.

**Additional file 2 Fig. S2.** Intragenic distribution of the AGO2 clusters within mRNAs.

**Additional file 3 Fig. S3.** Distribution of miR-124-3p AGO2-bound targets in the retina.

**Additional file 4 Table S1**. HTS data. **Table S2**. HTS miRNA data. **Table S3**. AGO2 HITS-CLIP mRNA data. **Table S5**. Significantly enriched pathways in global RNA between dim and PD retinas. **Table S6**. Up-regulated AGO2-bound mRNA. **Table S7**. Down-regulated AGO2-bound mRNA. **Table S8**. Up-regulated AGO2-bound mRNA GO – Molecular Function and Cellular Component. **Table S9**. Down-regulated AGO2-bound mRNA GO – Molecular Function and Cellular Component. **Table S10**. AGO2-bound mRNA predicted to bind to miR-124-3p.

## Data Availability

The datasets supporting the conclusions of this article are available on the Sequence Read Archive (PRJNA606092) as part of the National Center for Biotechnology Information (NCBI). Processed RNA-seq data can be interactively explored at: https://genedatasets.shinyapps.io/ARMMI/

## Abbreviations

3′ UTR: 3′ untranslated region
5′ UTR: 5′ untranslated region
AGO: argonaute
AGO2: argonaute 2
AMD: age-related macular degeneration
BWB: bead wash buffer
*Ccl2*: chemokine C-C motif ligand 2
CNS: central nervous system
CO_2_: carbon dioxide
DIG: digoxigenin
Dim: dim-reared
EGTA: ethylene glycol-bis(β-aminoethyl ether)-N,N,N′,N′-tetraacetic acid
GCL: ganglion cell layer
GO: gene ontology
GSEA: gene set enrichment analysis
HITS-CLIP: high-throughput sequencing following cross-linking immunoprecipitation
HTS: high-throughput sequencing
IgG: immunoglobulin G
INL: inner nuclear layer
LED: light-emitting diode
miRNA: microRNA
miRnome: miRNA transcriptome
mRNA: messenger RNA
ncRNA: non-coding RNA
OLM: outer limiting membrane
ONL: outer nuclear layer
PBS: phosphate buffered solution
PCA: principal component analysis
PCR: polymerase chain reaction
PD: photo-oxidative damage
PNK: polynucleotide kinase
RFP: red fluorescent protein
RGC: retinal ganglion cells
RIN: RNA integrity number
RISC: RNA-induced silencing complex
RNA: ribonucleic acid
*Rock2*: rho-associated protein kinase 2
RPM: revolutions per minute
RT: room temperature
scRNAseq: single-cell RNA sequencing
Targetome: target transcriptome
TTS: transcription termination site
UV: ultraviolet
