# Supplementary figures and images for: Functional microRNA targetome undergoes degeneration-induced shift in the retina
Source: Mol Neurodegener. 2021 Aug 31;16:60. doi: 10.1186/s13024-021-00478-9 (PMC8406976; doi:10.1186/s13024-021-00478-9)

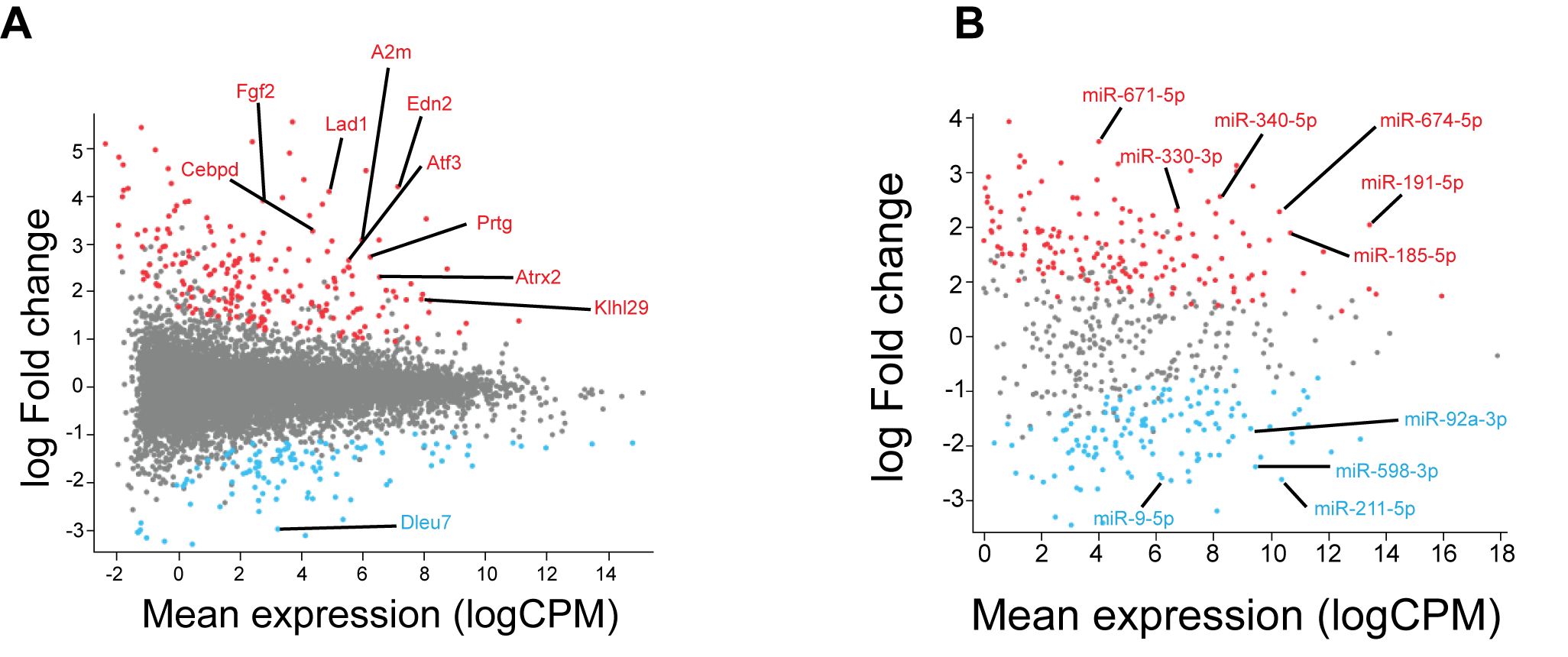

Supplement: Supplementary file 1 — Additional file 1 Fig. S1. Volcano plots of the differentially expressed mRNA (A) and miRNA (B) in the global retina dataset. [file 13024_2021_478_MOESM1_ESM.tif]

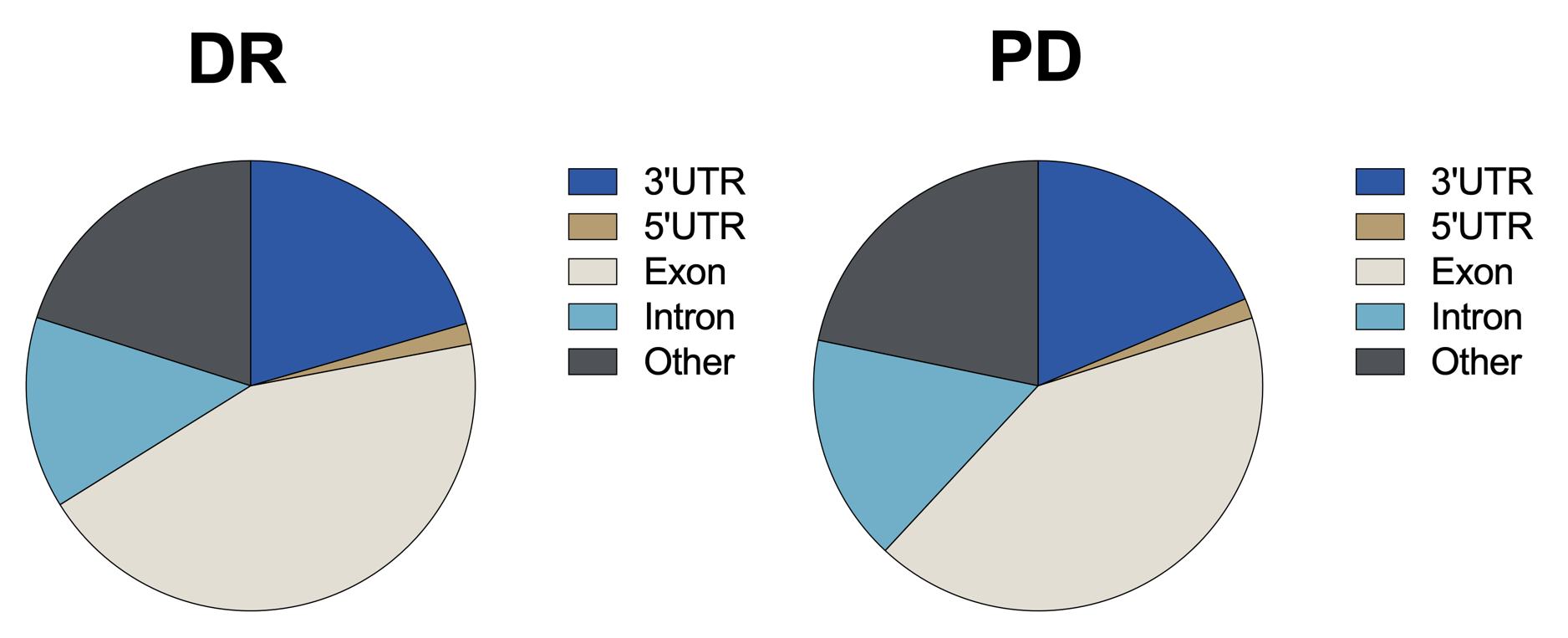

Supplement: Supplementary file 2 — Additional file 2 Fig. S2. Intragenic distribution of the AGO2 clusters within mRNAs. [file 13024_2021_478_MOESM2_ESM.tiff]

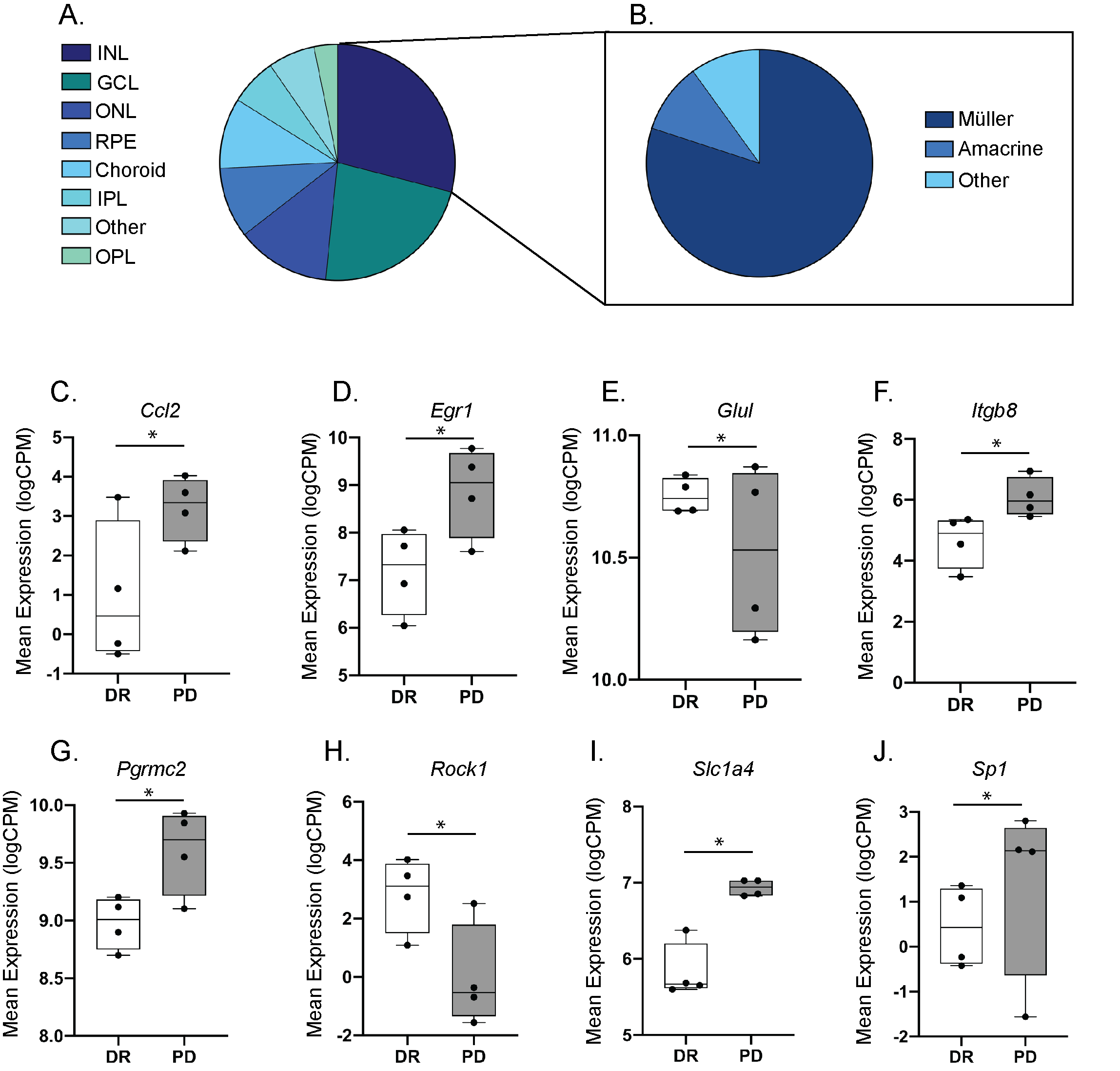

Supplement: Supplementary file 3 — Additional file 3 Fig. S3. Distribution of miR-124-3p AGO2-bound targets in the retina. [file 13024_2021_478_MOESM3_ESM.tif]
