# Supplementary material for: Functional microRNA targetome undergoes degeneration-induced shift in the retina
Source: Mol Neurodegener. 2021 Aug 31;16:60. doi: 10.1186/s13024-021-00478-9 (PMC8406976; doi:10.1186/s13024-021-00478-9)
Supplement: Supplementary file 4 — Additional file 4 Table S1. HTS data. Table S2. HTS miRNA data. Table S3. AGO2 HITS-CLIP mRNA data. Table S5. Significantly enriched pathways in global RNA between dim and PD retinas. Table S6. Up-regulated AGO2-bound mRNA. Table S7. Down-regulated AGO2-bound mRNA. Table S8. Up-regulated AGO2-bound mRNA GO – Molecular Function and Cellular Component. Table S9. Down-regulated AGO2-bound mRNA GO – Molecular Function and Cellular Component. Table S10. AGO2-bound mRNA predicted to bind to miR-124-3p. [file 13024_2021_478_MOESM4_ESM.docx]

**Supplementary tables**

Table S1. HTS data

| **Sample** | **Total Reads** | **Mapped (%)** |
| --- | --- | --- |
| DRT1 | 62183039 | 62006211 (99.7%) |
| DRT2 | 58695169 | 58527587 (99.7%) |
| DRT3 | 71704885 | 71506527 (99.7%) |
| DRT4 | 67294428 | 67082628 (99.7%) |
| PDT1 | 55833485 | 55682900 (99.7%) |
| PDT2 | 62055959 | 61883851 (99.7%) |
| PDT3 | 47402757 | 47259650 (99.7%) |
| PDT4 | 58157813 | 57998080 (99.7%) |

Table S2. HTS miRNA data

| **Sample** | **Total Reads** | **Mapped (%)** |
| --- | --- | --- |
| DRT1 | 9441386 | 475317 (5.03%) |
| DRT2 | 11634754 | 879886 (7.56%) |
| DRT3 | 13579093 | 450228 (3.32%) |
| DRT4 | 3401131 | 328870 (9.67%) |
| PDT1 | 5738275 | 670843 (11.7%) |
| PDT2 | 3788712 | 360037 (9.50%) |
| PDT3 | 7263393 | 496627 6.84%) |
| PDT4 | 9321032 | 573402 (6.15%) |

Table S3. AGO2 HITS-CLIP mRNA data

| **Sample** | **Total Reads** | **Mapped (%)** |
| --- | --- | --- |
| DR1 | 30000755 | 6307279 (21.0%) |
| DR2 | 26868648 | 5598812 (20.8%) |
| DR3 | 32838215 | 6316352 (19.2%) |
| DR4 | 30052795 | 6340151 (21.1%) |
| PD1 | 13578434 | 4808056 (35.4%) |
| PD2 | 29088557 | 9242024 (31.8%) |
| PD3 | 30009955 | 7628333 (25.4%) |
| PD4 | 24538691 | 5356323 (21.8%) |

Table S4. AGO2 HITS-CLIP miRNA data

| **Sample** | **Total Reads** | **Mapped (%)** |
| --- | --- | --- |
| DR1 | 5939381 | 5084500 (85.6%) |
| DR2 | 2426520 | 2066053 (85.1%) |
| DR3 | 6289561 | 5417244 (86.1%) |
| DR4 | 6934872 | 6029681 (86.9%) |
| PD1 | 4497423 | 3851689 (85.6%) |
| PD2 | 5495732 | 4703597 (85.6%) |
| PD3 | 6356439 | 5537296 (87.1%) |
| PD4 | 8936299 | 7785086 (87.1%) |

Table S5. Significantly enriched pathways in global RNA between dim and PD retinas

| **Pathway** | **Genes** | **Direction** | **P-value** | **FDR** |
| --- | --- | --- | --- | --- |
| PID RHODOPSIN PATHWAY | 19 | Down | 2.46E-12 | 1.16E-08 |
| BIOCARTA CLASSIC PATHWAY | 9 | Up | 9.93E-11 | 2.34E-07 |
| KUROZUMI RESPONSE TO ONCOCYTIC VIRUS | 13 | Up | 8.78E-10 | 1.10E-06 |
| NAKAYAMA SOFT TISSUE TUMORS PCA1 UP | 30 | Up | 9.33E-10 | 1.10E-06 |
| BIOCARTA COMP PATHWAY | 10 | Up | 1.36E-09 | 1.19E-06 |
| GAURNIER PSMD4 TARGETS | 37 | Up | 1.51E-09 | 1.19E-06 |
| WIELAND UP BY HBV INFECTION | 62 | Up | 5.41E-09 | 3.64E-06 |
| REACTOME INTERFERON ALPHA BETA SIGNALING | 29 | Up | 7.62E-09 | 4.49E-06 |
| KEGG ALLOGRAFT REJECTION | 18 | Up | 1.38E-08 | 7.23E-06 |
| ICHIBA GRAFT VERSUS HOST DISEASE D7 UP | 60 | Up | 1.79E-08 | 8.46E-06 |
| REACTOME INTERFERON GAMMA SIGNALING | 35 | Up | 2.26E-08 | 9.10E-06 |
| REACTOME ENDOSOMAL VACUOLAR PATHWAY | 10 | Up | 2.32E-08 | 9.10E-06 |
| KEGG GRAFT VERSUS HOST DISEASE | 19 | Up | 2.73E-08 | 9.73E-06 |
| SANA RESPONSE TO IFNG UP | 38 | Up | 2.89E-08 | 9.73E-06 |
| REACTOME IMMUNOREGULATORY INTERACTIONS BETWEEN A LYMPHOID AND A NON LYMPHOID CELL | 22 | Up | 3.38E-08 | 1.06E-05 |
| BIOCARTA CTL PATHWAY | 7 | Up | 4.78E-08 | 1.41E-05 |
| KIM LRRC3B TARGETS | 19 | Up | 5.94E-08 | 1.65E-05 |
| KEGG AUTOIMMUNE THYROID DISEASE | 20 | Up | 9.89E-08 | 2.59E-05 |
| ALTEMEIER RESPONSE TO LPS WITH MECHANICAL VENTILATION | 68 | Up | 1.08E-07 | 2.67E-05 |
| HECKER IFNB1 TARGETS | 44 | Up | 1.48E-07 | 3.49E-05 |
| PHONG TNF TARGETS UP | 37 | Up | 1.79E-07 | 3.94E-05 |
| MCLACHLAN DENTAL CARIES UP | 112 | Up | 1.84E-07 | 3.94E-05 |
| DAUER STAT3 TARGETS UP | 36 | Up | 2.95E-07 | 5.92E-05 |
| NAGASHIMA EGF SIGNALING UP | 44 | Up | 3.01E-07 | 5.92E-05 |
| MOSERLE IFNA RESPONSE | 14 | Up | 3.30E-07 | 6.22E-05 |
| REACTOME ANTIGEN PRESENTATION FOLDING ASSEMBLY AND PEPTIDE LOADING OF CLASS I MHC | 16 | Up | 3.52E-07 | 6.38E-05 |
| BROWNE INTERFERON RESPONSIVE GENES | 31 | Up | 5.31E-07 | 9.27E-05 |
| SCHOEN NFKB SIGNALING | 18 | Up | 7.58E-07 | 0.00012766 |
| ABBUD LIF SIGNALING 1 UP | 19 | Up | 9.66E-07 | 0.0001572 |
| SANA TNF SIGNALING UP | 47 | Up | 1.11E-06 | 0.00017436 |
| PID AP1 PATHWAY | 39 | Up | 1.30E-06 | 0.00019812 |
| REACTOME INITIAL TRIGGERING OF COMPLEMENT | 9 | Up | 1.38E-06 | 0.00020396 |
| UZONYI RESPONSE TO LEUKOTRIENE AND THROMBIN | 29 | Up | 1.49E-06 | 0.00021331 |
| SOUCEK MYC TARGETS | 6 | Up | 2.21E-06 | 0.0003072 |
| PID IL12 2PATHWAY | 20 | Up | 2.45E-06 | 0.00032619 |
| DEBOSSCHER NFKB TARGETS REPRESSED BY GLUCOCORTICOIDS | 6 | Up | 2.52E-06 | 0.00032619 |
| SEKI INFLAMMATORY RESPONSE LPS UP | 36 | Up | 2.56E-06 | 0.00032619 |
| KEGG TYPE I DIABETES MELLITUS | 26 | Up | 2.75E-06 | 0.00034157 |
| KEGG VIRAL MYOCARDITIS | 35 | Up | 3.29E-06 | 0.00038904 |
| MCLACHLAN DENTAL CARIES DN | 112 | Up | 3.30E-06 | 0.00038904 |
| YAN ESCAPE FROM ANOIKIS | 13 | Up | 3.39E-06 | 0.00038998 |
| KEGG ANTIGEN PROCESSING AND PRESENTATION | 36 | Up | 3.69E-06 | 0.00041484 |
| REACTOME NEF MEDIATED DOWNREGULATION OF MHC CLASS I COMPLEX CELL SURFACE EXPRESSION | 10 | Up | 3.84E-06 | 0.00042113 |
| BOSCO INTERFERON INDUCED ANTIVIRAL MODULE | 37 | Up | 4.34E-06 | 0.00046579 |
| AMIT EGF RESPONSE 20 HELA | 9 | Up | 4.95E-06 | 0.0005187 |
| KEGG COMPLEMENT AND COAGULATION CASCADES | 27 | Up | 5.09E-06 | 0.00052226 |
| CHASSOT SKIN WOUND | 7 | Up | 6.03E-06 | 0.0006054 |
| BURTON ADIPOGENESIS PEAK AT 2HR | 36 | Up | 6.23E-06 | 0.00061271 |
| PEDERSEN METASTASIS BY ERBB2 ISOFORM 1 | 26 | Up | 7.76E-06 | 0.00074708 |
| ICHIBA GRAFT VERSUS HOST DISEASE 35D UP | 76 | Up | 9.44E-06 | 0.00089092 |
| DER IFN GAMMA RESPONSE UP | 45 | Up | 9.84E-06 | 0.00089481 |
| DEMAGALHAES AGING UP | 39 | Up | 9.86E-06 | 0.00089481 |
| DER IFN BETA RESPONSE UP | 59 | Up | 1.03E-05 | 0.00091484 |
| JECHLINGER EPITHELIAL TO MESENCHYMAL TRANSITION DN | 39 | Up | 1.14E-05 | 0.00098775 |
| GRANDVAUX IFN RESPONSE NOT VIA IRF3 | 8 | Up | 1.15E-05 | 0.00098775 |
| LEE EARLY T LYMPHOCYTE DN | 29 | Up | 1.34E-05 | 0.00112503 |
| GESERICK TERT TARGETS DN | 15 | Up | 1.36E-05 | 0.00112503 |
| REACTOME COMPLEMENT CASCADE | 14 | Up | 1.39E-05 | 0.00112934 |
| CHEN ETV5 TARGETS SERTOLI | 10 | Up | 1.64E-05 | 0.0013018 |
| PID INTEGRIN2 PATHWAY | 12 | Up | 1.66E-05 | 0.0013018 |
| AMIT SERUM RESPONSE 40 MCF10A | 22 | Up | 1.80E-05 | 0.00137272 |
| FARMER BREAST CANCER CLUSTER 1 | 14 | Up | 1.80E-05 | 0.00137272 |
| LIAN LIPA TARGETS 3M | 28 | Up | 2.01E-05 | 0.00150663 |
| SCHURINGA STAT5A TARGETS DN | 7 | Up | 2.16E-05 | 0.00159184 |
| FLECHNER BIOPSY KIDNEY TRANSPLANT REJECTED VS OK UP | 45 | Up | 2.26E-05 | 0.00163688 |
| LIU VAV3 PROSTATE CARCINOGENESIS UP | 40 | Up | 2.33E-05 | 0.00166256 |
| AMIT DELAYED EARLY GENES | 14 | Up | 2.57E-05 | 0.00180673 |
| TIAN TNF SIGNALING VIA NFKB | 13 | Up | 2.65E-05 | 0.00183524 |
| BOYAULT LIVER CANCER SUBCLASS G5 DN | 10 | Up | 2.74E-05 | 0.00187447 |
| CHEOK RESPONSE TO HD MTX UP | 7 | Up | 3.06E-05 | 0.00204758 |
| AMIT EGF RESPONSE 40 MCF10A | 12 | Up | 3.08E-05 | 0.00204758 |
| BIOCARTA LAIR PATHWAY | 6 | Up | 3.19E-05 | 0.002089 |
| ZHANG INTERFERON RESPONSE | 10 | Up | 3.33E-05 | 0.00215104 |
| AMIT EGF RESPONSE 40 HELA | 28 | Up | 5.74E-05 | 0.00365022 |
| BIOCARTA DC PATHWAY | 3 | Up | 5.86E-05 | 0.00365022 |
| TSAI RESPONSE TO RADIATION THERAPY | 21 | Up | 5.88E-05 | 0.00365022 |
| RADAEVA RESPONSE TO IFNA1 UP | 29 | Up | 6.99E-05 | 0.00428494 |
| KUROKAWA LIVER CANCER EARLY RECURRENCE DN | 8 | Up | 7.14E-05 | 0.00431493 |
| DER IFN ALPHA RESPONSE UP | 43 | Up | 7.82E-05 | 0.00467177 |
| VERHAAK AML WITH NPM1 MUTATED UP | 83 | Up | 7.99E-05 | 0.00471304 |
| WALLACE PROSTATE CANCER RACE UP | 133 | Up | 8.29E-05 | 0.00482962 |
| MCDOWELL ACUTE LUNG INJURY UP | 29 | Up | 8.62E-05 | 0.0049564 |
| REACTOME REGULATION OF COMPLEMENT CASCADE | 8 | Up | 9.36E-05 | 0.00532136 |
| SA MMP CYTOKINE CONNECTION | 5 | Up | 9.65E-05 | 0.00541711 |
| HESS TARGETS OF HOXA9 AND MEIS1 DN | 35 | Up | 9.77E-05 | 0.0054214 |
| KUROZUMI RESPONSE TO ONCOCYTIC VIRUS AND CYCLIC RGD | 3 | Up | 9.90E-05 | 0.00542806 |
| REACTOME INTERFERON SIGNALING | 93 | Up | 0.00011007 | 0.00596802 |
| PID CONE PATHWAY | 18 | Down | 0.00012825 | 0.00681899 |
| VANHARANTA UTERINE FIBROID DN | 35 | Up | 0.00012866 | 0.00681899 |
| APPEL IMATINIB RESPONSE | 15 | Up | 0.00013229 | 0.00693362 |
| STAMBOLSKY TARGETS OF MUTATED TP53 DN | 24 | Up | 0.00014255 | 0.00732291 |
| KHETCHOUMIAN TRIM24 TARGETS UP | 29 | Up | 0.00014283 | 0.00732291 |
| MARIADASON RESPONSE TO BUTYRATE CURCUMIN SULINDAC TSA 1 | 6 | Up | 0.00015206 | 0.00771275 |
| PID CD8 TCR DOWNSTREAM PATHWAY | 26 | Up | 0.00015944 | 0.00795148 |
| BIOCARTA RANKL PATHWAY | 10 | Up | 0.00016014 | 0.00795148 |
| EINAV INTERFERON SIGNATURE IN CANCER | 15 | Up | 0.00017957 | 0.00882324 |
| UROSEVIC RESPONSE TO IMIQUIMOD | 5 | Up | 0.00020462 | 0.00982623 |
| VILIMAS NOTCH1 TARGETS UP | 22 | Up | 0.00020523 | 0.00982623 |
| KOBAYASHI EGFR SIGNALING 6HR DN | 12 | Up | 0.00020652 | 0.00982623 |
| BIOCARTA LYM PATHWAY | 5 | Up | 0.00021033 | 0.00982623 |
| BENNETT SYSTEMIC LUPUS ERYTHEMATOSUS | 14 | Up | 0.0002104 | 0.00982623 |
| MORI LARGE PRE BII LYMPHOCYTE DN | 20 | Up | 0.00023339 | 0.01069883 |
| VALK AML CLUSTER 5 | 14 | Up | 0.00023362 | 0.01069883 |
| WUNDER INFLAMMATORY RESPONSE AND CHOLESTEROL UP | 29 | Up | 0.00024001 | 0.0108096 |
| AMIT EGF RESPONSE 60 MCF10A | 26 | Up | 0.00024062 | 0.0108096 |
| KEGG LEISHMANIA INFECTION | 38 | Up | 0.00024524 | 0.0109133 |
| VERHAAK GLIOBLASTOMA MESENCHYMAL | 94 | Up | 0.00025052 | 0.01104376 |
| LIAN LIPA TARGETS 6M | 37 | Up | 0.00025965 | 0.01134023 |
| FERRANDO TAL1 NEIGHBORS | 8 | Up | 0.00026341 | 0.01139901 |
| BIOCARTA LECTIN PATHWAY | 5 | Up | 0.00026742 | 0.01146727 |
| GHANDHI BYSTANDER IRRADIATION UP | 42 | Up | 0.00030082 | 0.01268463 |
| BURTON ADIPOGENESIS 7 | 25 | Up | 0.00030118 | 0.01268463 |
| REACTOME GENERATION OF SECOND MESSENGER MOLECULES | 6 | Up | 0.00030585 | 0.01276733 |
| PID INTEGRIN CS PATHWAY | 12 | Up | 0.00030869 | 0.01277255 |
| WU HBX TARGETS 2 DN | 10 | Up | 0.00032432 | 0.01330256 |
| WIEDERSCHAIN TARGETS OF BMI1 AND PCGF2 | 35 | Up | 0.00033187 | 0.01349518 |
| TONKS TARGETS OF RUNX1 RUNX1T1 FUSION ERYTHROCYTE UP | 85 | Up | 0.00034807 | 0.01403299 |
| CHEN HOXA5 TARGETS 6HR UP | 7 | Up | 0.00035912 | 0.01435549 |
| DAZARD UV RESPONSE CLUSTER G28 | 10 | Up | 0.00036386 | 0.01442308 |
| KEGG INTESTINAL IMMUNE NETWORK FOR IGA PRODUCTION | 20 | Up | 0.00038279 | 0.01504684 |
| KIM GLIS2 TARGETS UP | 55 | Up | 0.00043878 | 0.01707191 |
| HAHTOLA MYCOSIS FUNGOIDES CD4 UP | 36 | Up | 0.00044492 | 0.01707191 |
| TAKEDA TARGETS OF NUP98 HOXA9 FUSION 3D UP | 84 | Up | 0.00044517 | 0.01707191 |
| WINZEN DEGRADED VIA KHSRP | 52 | Up | 0.00047361 | 0.01801615 |
| DIRMEIER LMP1 RESPONSE EARLY | 35 | Up | 0.00049628 | 0.01872777 |
| BROWN MYELOID CELL DEVELOPMENT UP | 85 | Up | 0.00050995 | 0.01889873 |
| PID IL6 7 PATHWAY | 24 | Up | 0.00051168 | 0.01889873 |
| STEARMAN TUMOR FIELD EFFECT UP | 17 | Up | 0.00051283 | 0.01889873 |
| REACTOME NEF MEDIATES DOWN MODULATION OF CELL SURFACE RECEPTORS BY RECRUITING THEM TO CLATHRIN ADAPTERS | 14 | Up | 0.0005231 | 0.01912765 |
| WILENSKY RESPONSE TO DARAPLADIB | 17 | Up | 0.00054127 | 0.01945705 |
| REACTOME CYTOKINE SIGNALING IN IMMUNE SYSTEM | 142 | Up | 0.00054468 | 0.01945705 |
| MAHADEVAN RESPONSE TO MP470 UP | 11 | Up | 0.00054798 | 0.01945705 |
| OUYANG PROSTATE CANCER PROGRESSION UP | 12 | Up | 0.00054861 | 0.01945705 |
| WUNDER INFLAMMATORY RESPONSE AND CHOLESTEROL DN | 9 | Up | 0.00055332 | 0.01947764 |
| KANG GIST WITH PDGFRA UP | 33 | Up | 0.00056534 | 0.01975326 |
| KEGG CELL ADHESION MOLECULES CAMS | 66 | Up | 0.00058462 | 0.02027696 |
| HALMOS CEBPA TARGETS UP | 26 | Up | 0.00062591 | 0.02155056 |
| KEGG ASTHMA | 7 | Up | 0.0006553 | 0.02239903 |
| TURJANSKI MAPK7 TARGETS | 2 | Up | 0.00066205 | 0.02245393 |
| REACTOME THE ROLE OF NEF IN HIV1 REPLICATION AND DISEASE PATHOGENESIS | 16 | Up | 0.00067045 | 0.02245393 |
| SEITZ NEOPLASTIC TRANSFORMATION BY 8P DELETION UP | 36 | Up | 0.00067119 | 0.02245393 |
| HAN JNK SINGALING UP | 21 | Up | 0.00067983 | 0.02258287 |
| REACTOME CREATION OF C4 AND C2 ACTIVATORS | 5 | Up | 0.00069002 | 0.02264095 |
| KEGG NATURAL KILLER CELL MEDIATED CYTOTOXICITY | 48 | Up | 0.00069118 | 0.02264095 |
| WIERENGA STAT5A TARGETS GROUP2 | 34 | Up | 0.00069735 | 0.0226854 |
| LEE AGING CEREBELLUM UP | 54 | Up | 0.00070479 | 0.02273283 |
| KEGG PRION DISEASES | 21 | Up | 0.00070844 | 0.02273283 |
| DAUER STAT3 TARGETS DN | 30 | Up | 0.00071708 | 0.02285452 |
| BIOCARTA NKCELLS PATHWAY | 12 | Up | 0.00077424 | 0.02451081 |
| GRAHAM CML QUIESCENT VS NORMAL QUIESCENT DN | 25 | Up | 0.00079424 | 0.02497617 |
| LINDSTEDT DENDRITIC CELL MATURATION A | 26 | Up | 0.00082857 | 0.02588333 |
| RASHI RESPONSE TO IONIZING RADIATION 2 | 71 | Up | 0.00083496 | 0.02591107 |
| TOMLINS METASTASIS DN | 15 | Up | 0.00087331 | 0.0269242 |
| LIU VAV3 PROSTATE CARCINOGENESIS DN | 14 | Up | 0.00088473 | 0.02699929 |
| PHONG TNF RESPONSE VIA P38 PARTIAL | 96 | Up | 0.00089671 | 0.02699929 |
| PID FRA PATHWAY | 16 | Up | 0.00089737 | 0.02699929 |
| LINDGREN BLADDER CANCER CLUSTER 2B | 218 | Up | 0.00089864 | 0.02699929 |
| GALINDO IMMUNE RESPONSE TO ENTEROTOXIN | 55 | Up | 0.00092154 | 0.02751196 |
| ZIRN TRETINOIN RESPONSE WT1 DN | 1 | Down | 0.00095136 | 0.02816017 |
| TONKS TARGETS OF RUNX1 RUNX1T1 FUSION SUSTAINDED IN ERYTHROCYTE UP | 26 | Up | 0.00095519 | 0.02816017 |
| SARTIPY NORMAL AT INSULIN RESISTANCE UP | 23 | Up | 0.00096951 | 0.02840479 |
| BOYAULT LIVER CANCER SUBCLASS G56 DN | 10 | Up | 0.00102584 | 0.0298696 |
| MAHADEVAN RESPONSE TO MP470 DN | 15 | Up | 0.00103256 | 0.02988087 |
| SASAKI TARGETS OF TP73 AND TP63 | 6 | Up | 0.00105493 | 0.03034221 |
| HADDAD T LYMPHOCYTE AND NK PROGENITOR DN | 32 | Up | 0.00106139 | 0.03034301 |
| NEMETH INFLAMMATORY RESPONSE LPS UP | 58 | Up | 0.00108603 | 0.03085683 |
| NOJIMA SFRP2 TARGETS DN | 10 | Up | 0.00109686 | 0.03085683 |
| BIOCARTA MONOCYTE PATHWAY | 3 | Up | 0.00109899 | 0.03085683 |
| BORLAK LIVER CANCER EGF UP | 37 | Up | 0.00113218 | 0.03146267 |
| IIZUKA LIVER CANCER PROGRESSION L0 L1 DN | 19 | Up | 0.00113391 | 0.03146267 |
| MIZUKAMI HYPOXIA UP | 7 | Up | 0.00114527 | 0.03159216 |
| LEE LIVER CANCER MYC E2F1 UP | 32 | Up | 0.00116908 | 0.03206132 |
| BERENJENO ROCK SIGNALING NOT VIA RHOA UP | 17 | Up | 0.00119566 | 0.0325492 |
| ABRAHAM ALPC VS MULTIPLE MYELOMA UP | 14 | Up | 0.00120067 | 0.0325492 |
| ALONSO METASTASIS EMT DN | 2 | Up | 0.0013303 | 0.03585741 |
| BASSO CD40 SIGNALING UP | 52 | Up | 0.00136525 | 0.03659014 |
| AMIT SERUM RESPONSE 120 MCF10A | 41 | Up | 0.00137704 | 0.03669774 |
| GRANDVAUX IRF3 TARGETS UP | 7 | Up | 0.0014144 | 0.03748162 |
| HERNANDEZ MITOTIC ARREST BY DOCETAXEL 2 DN | 7 | Up | 0.00144438 | 0.03792119 |
| BURTON ADIPOGENESIS 9 | 59 | Up | 0.00144707 | 0.03792119 |
| REACTOME INNATE IMMUNE SYSTEM | 106 | Up | 0.00145746 | 0.03798245 |
| LIANG SILENCED BY METHYLATION 2 | 24 | Up | 0.00148188 | 0.0383216 |
| TAVOR CEBPA TARGETS UP | 30 | Up | 0.00148672 | 0.0383216 |
| ODONNELL TARGETS OF MYC AND TFRC UP | 46 | Up | 0.00149879 | 0.03832402 |
| JACKSON DNMT1 TARGETS UP | 43 | Up | 0.00150306 | 0.03832402 |
| AMIT EGF RESPONSE 60 HELA | 31 | Up | 0.00156173 | 0.0396057 |
| ONO AML1 TARGETS UP | 12 | Up | 0.0015959 | 0.04025605 |
| WATTEL AUTONOMOUS THYROID ADENOMA DN | 28 | Up | 0.00162163 | 0.04068749 |
| SCHUETZ BREAST CANCER DUCTAL INVASIVE UP | 174 | Up | 0.00164318 | 0.04085975 |
| CHIARADONNA NEOPLASTIC TRANSFORMATION KRAS CDC25 DN | 33 | Up | 0.00164582 | 0.04085975 |
| HUMMERICH SKIN CANCER PROGRESSION UP | 53 | Up | 0.00170384 | 0.04207851 |
| BIOCARTA IL6 PATHWAY | 13 | Up | 0.0018368 | 0.04512589 |
| BIOCARTA DREAM PATHWAY | 10 | Up | 0.00185482 | 0.04519027 |
| BROCKE APOPTOSIS REVERSED BY IL6 | 90 | Up | 0.00185858 | 0.04519027 |
| KRASNOSELSKAYA ILF3 TARGETS UP | 18 | Up | 0.00188503 | 0.0454168 |
| PLASARI TGFB1 TARGETS 1HR UP | 21 | Up | 0.00188715 | 0.0454168 |
| LIAN NEUTROPHIL GRANULE CONSTITUENTS | 7 | Up | 0.00196019 | 0.04693508 |
| GUENTHER GROWTH SPHERICAL VS ADHERENT DN | 15 | Up | 0.00199556 | 0.04732302 |
| ST GRANULE CELL SURVIVAL PATHWAY | 13 | Up | 0.00199646 | 0.04732302 |
| ZAIDI OSTEOBLAST TRANSCRIPTION FACTORS | 7 | Up | 0.00202758 | 0.04771888 |
| REACTOME SIGNAL REGULATORY PROTEIN SIRP FAMILY INTERACTIONS | 3 | Up | 0.00203339 | 0.04771888 |
| BERENJENO TRANSFORMED BY RHOA REVERSIBLY DN | 16 | Up | 0.00212021 | 0.04951006 |

Table S6. Up-regulated AGO2-bound mRNA

| **Gene** | **logFold Change in PD vs dim** | **Adjusted P-value** |
| --- | --- | --- |
| *Prtg* | 2.21 | 1.00E-05 |
| *Antxr2* | 2.36 | 1.53E-05 |
| *Edn2* | 5.46 | 1.53E-05 |
| *Myo10* | 1.80 | 2.29E-05 |
| *Trf* | 2.59 | 9.86E-05 |
| *A2m* | 5.23 | 0.000111 |
| *Slc1a1* | 1.82 | 0.000142 |
| *Atp1a1* | 1.02 | 0.000208 |
| *Lad1* | 4.03 | 0.000208 |
| *Cebpd* | 4.44 | 0.000388 |
| *Klhl29* | 1.55 | 0.000388 |
| *Il6st* | 1.18 | 0.00103 |
| *Junb* | 2.92 | 0.00219 |
| *Gnb3* | 1.25 | 0.00344 |
| *Gadd45b* | 2.66 | 0.00390 |
| *Lcn2* | 4.54 | 0.00441 |
| *Adamts1* | 1.84 | 0.00500 |
| *Tgm2* | 4.01 | 0.00511 |
| *Map3k1* | 1.10 | 0.00561 |
| *Rsad2* | 3.54 | 0.00953 |
| *Agtpbp1* | 1.67 | 0.0114 |
| *Gm41608* | 3.46 | 0.0114 |
| *Ifit1* | 3.79 | 0.0114 |
| *H2-Q4* | 3.63 | 0.0120 |
| *Hey2* | 1.30 | 0.0120 |
| *H2-D1* | 1.53 | 0.0132 |
| *Lgals3bp* | 1.62 | 0.0136 |
| *Plpp3* | 0.90 | 0.0149 |
| *Egr1* | 1.91 | 0.0175 |
| *Phlpp2* | 0.59 | 0.0175 |
| *Itga4* | 1.01 | 0.0177 |
| *Zcchc24* | 0.91 | 0.0180 |
| *Slc1a4* | 1.16 | 0.0218 |
| *Atf3* | 2.93 | 0.0218 |
| *Ifitm3* | 2.12 | 0.0246 |
| *Mt2* | 2.85 | 0.0291 |
| *Syngr3* | 1.28 | 0.0296 |
| *B2m* | 2.50 | 0.0297 |
| *R3hcc1l* | 0.89 | 0.0297 |
| *Tor3a* | 1.36 | 0.0297 |
| *Crym* | 1.56 | 0.0393 |
| *ND1* | 0.62 | 0.0411 |
| *Sox9* | 0.93 | 0.0472 |

Table S7. Down-regulated AGO2-bound mRNA

| **Gene** | **logFold Change in PD vs dim** | **Adjusted P-value** |
| --- | --- | --- |
| *Gnat1* | -1.48 | 3.53E-06 |
| *Rho* | -1.94 | 1.00E-05 |
| *Prph2* | -1.32 | 1.53E-05 |
| *Pde6b* | -1.08 | 1.84E-05 |
| *Rom1* | -1.26 | 3.93E-05 |
| *Aqp1* | -1.74 | 8.40E-05 |
| *Nt5e* | -1.36 | 0.000151 |
| *BB283400* | -1.26 | 0.000201 |
| *Rgs9bp* | -0.94 | 0.000208 |
| *Rbp3* | -0.68 | 0.000208 |
| *Gucy2f* | -1.24 | 0.000388 |
| *Pde6a* | -0.88 | 0.00117 |
| *Pdzph1* | -1.16 | 0.00117 |
| *Nxnl1* | -1.41 | 0.00123 |
| *Rp1l1* | -0.80 | 0.00127 |
| *Grk1* | -0.81 | 0.00128 |
| *Guca1b* | -1.01 | 0.00209 |
| *Rs1* | -1.05 | 0.00245 |
| *Arl4d* | -1.31 | 0.00385 |
| *Abcg1* | -0.75 | 0.00441 |
| *Slc9a7* | -0.97 | 0.00511 |
| *Vtn* | -0.91 | 0.00535 |
| *Stk35* | -0.94 | 0.00793 |
| *Ammecr1* | -0.99 | 0.00979 |
| *Slc17a7* | -0.76 | 0.0108 |
| *LOC102634835* | -2.01 | 0.0108 |
| *Plekhf2* | -0.88 | 0.0114 |
| *Tmem170b* | -0.68 | 0.0114 |
| *Lbhd1* | -1.12 | 0.0114 |
| *Slc16a1* | -0.83 | 0.0129 |
| *Eno1b* | -0.87 | 0.0155 |
| *Acadvl* | -0.96 | 0.0156 |
| *Fam57b* | -1.46 | 0.0158 |
| *Pfkfb2* | -0.87 | 0.0158 |
| *Pgk1* | -1.03 | 0.01703 |
| *Aldoart1* | -1.36 | 0.0173 |
| *Kcnv2* | -0.99 | 0.0175 |
| *Klkb1* | -0.73 | 0.0176 |
| *Ypel2* | -0.53 | 0.0179 |
| *Ccdc24* | -3.73 | 0.0217 |
| *Hsp90aa1* | -0.56 | 0.0218 |
| *Hist1h1c* | -1.83 | 0.0218 |
| *Me2* | -0.95 | 0.0284 |
| *Cngb1* | -0.97 | 0.0291 |
| *AI847159* | -1.46 | 0.0292 |
| *Hk2* | -1.29 | 0.0292 |
| *Rcvrn* | -0.86 | 0.0297 |
| *Dleu7* | -3.28 | 0.0405 |
| *Esrrb* | -1.50 | 0.0415 |

Table S8. Up-regulated AGO2-bound mRNA GO – Molecular Function and Cellular Component

| **GO term** | **Description** | **P-value** | **FDR** |
| --- | --- | --- | --- |
| GO:0030881 | beta-2-microglobulin binding | 4.41E-08 | 1.46E-04 |
| GO:0042610 | CD8 receptor binding | 4.50E-07 | 7.45E-04 |
| GO:0042605 | peptide antigen binding | 6.92E-07 | 7.64E-04 |
| GO:0042608 | T cell receptor binding | 8.02E-07 | 6.64E-04 |
| GO:0062061 | TAP complex binding | 1.37E-06 | 9.10E-04 |
| GO:0042802 | identical protein binding | 4.19E-06 | 2.31E-03 |
| GO:0046977 | TAP binding | 1.04E-05 | 4.94E-03 |
| GO:0003823 | antigen binding | 2.31E-05 | 9.57E-03 |
| GO:0016755 | transferase activity, transferring amino-acyl groups | 1.70E-04 | 6.26E-02 |
| GO:0019959 | interleukin-8 binding | 2.77E-04 | 9.18E-02 |
| GO:0019966 | interleukin-1 binding | 2.77E-04 | 8.35E-02 |
| GO:0060089 | molecular transducer activity | 2.88E-04 | 7.93E-02 |
| GO:0004896 | cytokine receptor activity | 3.31E-04 | 8.44E-02 |
| GO:0015194 | L-serine transmembrane transporter activity | 3.33E-04 | 7.87E-02 |
| GO:0031708 | endothelin B receptor binding | 3.37E-04 | 7.43E-02 |
| GO:0038023 | signaling receptor activity | 3.60E-04 | 7.45E-02 |
| GO:1903981 | enterobactin binding | 4.16E-04 | 8.10E-02 |
| GO:0097677 | STAT family protein binding | 4.61E-04 | 8.48E-02 |
| GO:0004888 | transmembrane signaling receptor activity | 5.43E-04 | 9.46E-02 |
| GO:0010385 | double-stranded methylated DNA binding | 6.79E-04 | 1.12E-01 |
| GO:0015171 | amino acid transmembrane transporter activity | 6.98E-04 | 1.10E-01 |
| GO:0098748 | endocytic adaptor activity | 7.04E-04 | 1.06E-01 |
| GO:0035615 | clathrin adaptor activity | 7.04E-04 | 1.01E-01 |
| GO:0003810 | protein-glutamine gamma-glutamyltransferase activity | 8.32E-04 | 1.15E-01 |
| GO:0015175 | neutral amino acid transmembrane transporter activity | 8.91E-04 | 1.18E-01 |
| GO:0005515 | protein binding | 9.08E-04 | 1.16E-01 |
| GO:0008514 | organic anion transmembrane transporter activity | 9.79E-04 | 1.20E-01 |
| GO:0042611 | MHC protein complex | 5.50E-10 | 9.12E-07 |
| GO:0042612 | MHC class I protein complex | 6.25E-10 | 5.18E-07 |
| GO:0044421 | extracellular region part | 5.85E-09 | 3.23E-06 |
| GO:0005615 | extracellular space | 2.47E-08 | 1.02E-05 |
| GO:0042824 | MHC class I peptide loading complex | 1.66E-06 | 5.51E-04 |
| GO:0005576 | extracellular region | 2.18E-06 | 6.01E-04 |
| GO:0098552 | side of membrane | 7.32E-06 | 1.73E-03 |
| GO:0009897 | external side of plasma membrane | 9.05E-06 | 1.88E-03 |
| GO:0070971 | endoplasmic reticulum exit site | 8.77E-05 | 1.62E-02 |
| GO:1990712 | HFE-transferrin receptor complex | 1.30E-04 | 2.16E-02 |
| GO:0009986 | cell surface | 1.70E-04 | 2.56E-02 |
| GO:0098797 | plasma membrane protein complex | 2.58E-04 | 3.57E-02 |
| GO:0098802 | plasma membrane receptor complex | 2.85E-04 | 3.64E-02 |
| GO:0030670 | phagocytic vesicle membrane | 3.45E-04 | 4.09E-02 |
| GO:0005797 | Golgi medial cisterna | 4.80E-04 | 5.31E-02 |
| GO:0001939 | female pronucleus | 5.86E-04 | 6.07E-02 |
| GO:0044217 | other organism part | 8.52E-04 | 8.31E-02 |

Table S9. Down-regulated AGO2-bound mRNA GO – Molecular Function and Cellular Component

| **GO term** | **Description** | **P-value** | **FDR q-value** |
| --- | --- | --- | --- |
| GO:0048018 | receptor ligand activity | 4.43E-06 | 1.46E-02 |
| GO:0030545 | receptor regulator activity | 1.01E-05 | 1.67E-02 |
| GO:0017124 | SH3 domain binding | 5.90E-04 | 6.51E-01 |
| GO:0000776 | kinetochore | 2.95E-04 | 4.89E-01 |
| GO:0001750 | photoreceptor outer segment | 3.79E-04 | 3.14E-01 |
| GO:0001917 | photoreceptor inner segment | 3.88E-04 | 2.14E-01 |
| GO:0000775 | chromosome, centromeric region | 5.24E-04 | 2.17E-01 |
| GO:0005576 | extracellular region | 7.77E-04 | 2.58E-01 |

Table S10. AGO2-bound mRNA predicted to bind to miR-124-3p

| **Gene** | **logFold Change in PD vs dim** | **P-value** |
| --- | --- | --- |
| *Antxr2* | 2.3626328 | 9.70E-09 |
| *Myo10* | 1.798979023 | 1.95E-08 |
| *Stk35* | -0.939450988 | 3.55E-05 |
| *Ammecr1* | -0.994602386 | 4.59E-05 |
| *Plekhf2* | -0.876329158 | 5.98E-05 |
| *Slc16a1* | -0.829380386 | 7.58E-05 |
| *Egr1* | 1.90922451 | 0.000124661 |
| *Zcchc24* | 0.914702034 | 0.000136088 |
| *Slc1a4* | 1.160994865 | 0.000177268 |
| *Tor3a* | 1.363762931 | 0.00027897 |
| *Slc2a13* | 0.891574534 | 0.000858168 |
| *Acaa2* | 1.114278205 | 0.000860691 |
| *Fam199x* | -0.506414329 | 0.002787212 |
| *Tob2* | -0.497194078 | 0.002837396 |
| *Ccl2* | 2.711785452 | 0.00352564 |
| *Glul* | -0.408204948 | 0.004504003 |
| *Thrb* | -2.493894838 | 0.007497924 |
| *Fam53b* | -2.113053204 | 0.007877904 |
| *Ccdc117* | -0.505556285 | 0.009372384 |
| *Anxa5* | 0.79985392 | 0.011338264 |
| *Itgb8* | 1.124728236 | 0.011993412 |
| *Fam89a* | -0.797404686 | 0.012763135 |
| *Xkr4* | 0.638058956 | 0.013658401 |
| *Ascc2* | 0.683486851 | 0.01366163 |
| *Klf6* | 0.624593326 | 0.01662975 |
| *Lonrf1* | 0.444487282 | 0.016767637 |
| *Nbeal2* | 0.795824543 | 0.017171796 |
| *Pgrmc2* | 0.440415689 | 0.018366563 |
| *Tiprl* | -2.140248833 | 0.019541074 |
| *Rgs9* | -0.33650089 | 0.021885195 |
| *Klhl28* | -0.614793382 | 0.025672891 |
| *Slc7a8* | 0.687754313 | 0.026484867 |
| *Pik3ca* | -1.808157219 | 0.026708444 |
| *Tmcc3* | 1.986152996 | 0.029708159 |
| *Spry3* | -0.501623413 | 0.029870207 |
| *Nr1d2* | -0.354795692 | 0.030021147 |
| *Rnf11* | -0.365613023 | 0.03078371 |
| *Lrrc58* | 0.219233176 | 0.031143983 |
| *Sigmar1* | -0.624054103 | 0.033853882 |
| *Rock2* | 0.882579372 | 0.035479855 |
| *Ncoa2* | -2.043173774 | 0.036152073 |
| *Srsf6* | 0.482334496 | 0.039718188 |
| *Rock1* | -2.158513569 | 0.039728568 |
| *Mmp16* | 0.973148135 | 0.039863984 |
| *B4galt6* | 0.387926472 | 0.040073129 |
| *Rbm20* | -2.00730089 | 0.040365883 |
| *Myh9* | 0.288650483 | 0.0407971 |
| *Sptlc2* | 0.37871085 | 0.040926428 |
| *Dusp6* | 0.944425222 | 0.041557607 |
| *Nr3c1* | -1.757589789 | 0.042048432 |
| *Ttc9* | 0.420767978 | 0.04213689 |
| *Mapre1* | 0.329427138 | 0.042708551 |
| *Arih1* | 0.715195672 | 0.044913444 |
| *Oxsr1* | -2.063965828 | 0.045342592 |
| *Sp1* | 1.834869082 | 0.045361948 |
| *Jag1* | -1.171294072 | 0.045984879 |
| *Ncor2* | 1.879010576 | 0.047358544 |
| *Tub* | -0.30229807 | 0.047541267 |
| *Sertad3* | 0.815671744 | 0.048025558 |
| *Stt3a* | -0.342667898 | 0.048466343 |
| *Csgalnact2* | 0.942186647 | 0.048469156 |
